# Development of truncated elastin-like peptide analogues with improved temperature-response and self-assembling properties

**DOI:** 10.1038/s41598-022-23940-0

**Published:** 2022-11-12

**Authors:** Shogo Sumiyoshi, Keitaro Suyama, Naoki Tanaka, Takumi Andoh, Akihiko Nagata, Keisuke Tomohara, Suguru Taniguchi, Iori Maeda, Takeru Nose

**Affiliations:** 1 Laboratory of Biomolecular Chemistry, Department of Chemistry, Faculty and Graduate School of Science, Kyushu University, Fukuoka, 819-0395 Japan; 2 Laboratory of Biomolecular Chemistry, Faculty of Arts and Science, Kyushu University, Fukuoka, 819-0395 Japan; 3 Department of Physics and Information Technology, Kyushu Institute of Technology, Iizuka, Fukuoka 820-8502 Japan; 4 Present Address: Division of Biomedical Sciences, Fukuoka Dental College, Fukuoka, 814-0193 Japan

**Keywords:** Peptides, Pollution remediation

## Abstract

Functional peptides, which are composed of proteinogenic natural amino acids, are expected to be used as biomaterials with minimal environmental impact. Synthesizing a functional peptide with a shorter amino acid sequence while retaining its function is a easy and economical strategy. Furthermore, shortening functional peptides helps to elucidate the mechanism of their functional core region. Truncated elastin-like peptides (ELPs) are peptides consisting of repetitive sequences, derived from the elastic protein tropoelastin, that show the thermosensitive formation of coacervates. In this study, to obtain shortened ELP analogues, we synthesized several (Phe-Pro-Gly-Val-Gly)_n_ (FPGVG)_n_ analogues with one or two amino acid residues deleted from each repeat sequence, such as the peptide analogues consisting of FPGV and/or FPG sequences. Among the novel truncated ELP analogues, the 16-mer (FPGV)_4_ exhibited a stronger coacervation ability than the 25-mer (FPGVG)_5_. These results indicated that the coacervation ability of truncated ELPs was affected by the amino acid sequence and not by the peptide chain length. Based on this finding, we prepared Cd^2+^-binding sequence-conjugated ELP analogue, AADAAC-(FPGV)_4_, and found that it could capture Cd^2+^. These results indicated that the 16-mer (FPGV)_4_ only composed of proteinogenic amino acids could be a new biomaterial with low environmental impact.

## Introduction

To solve the problem of the huge accumulation of plastic waste today, the world is shifting from using petrochemical-derived materials to using eco-friendly biodegradable materials in various manufacturing industries^1,2^. Peptides and proteins, composed of proteinaceous amino acids, are typical examples of environmentally friendly biodegradable materials^3–5^, which may help solve the problem of plastic waste accumulation in environmental water. Despite their environmentally friendly properties, proteins and peptides are not extensively used as materials because proteins are labor-intensive to express, purify, and store, and long peptides are expensive to synthesize. One cost-saving approach is to reduce the number of amino acid residues required for the desired peptide properties. In addition, shortening functional peptides and proteins is not only economical but also has the advantage of improving safety by removing antigenic parts and enhancing function by selectively extracting the regions required for activity and function^6–9^.

Elastin-like peptides (ELPs) consist of repetitive internal sequences naturally found in tropoelastin, a representative elastic protein that provides resilience and elasticity to elastic tissues and organs^10^. ELPs are stimuli-responsive biomaterials that exhibit temperature-dependent reversible self-assembly, known as coacervation, under physiological conditions. ELPs, environmentally and biologically friendly biomaterials composed only of natural amino acids, have attracted attention owing to their potential applications as drug delivery systems^11–14^, metal scavengers^15–17^, and protein separation supports^18–20^. Among the various elastin-derived sequences, Val-Pro-Gly-Val-Gly (VPGVG) is a representative sequence that has been widely utilized in various scientific and applied studies^21–23^. However, owing to the weak coacervation potency of the VPGVG-based repetitive sequence, ELPs with long chains of more than 200 amino acid residues have mainly been used in applied research^24–27^. In recent years, progress has been achieved in the development of ELPs with shorter amino acid chain lengths; in particular, the substitution of hydrophobic amino acids in ELPs has led to the development of analogues that exhibit coacervation in relatively short chains. We have developed the ELP analogues H-(Phe-Pro-Gly-Val-Gly)_n_–OH, (FPGVG)_n_, which are shorter and have stronger coacervation ability compared with that of (VPGVG)_n_^28^. Notably, although (FPGVG)_5_ has only 25 amino acid residues, it showed apparent reversible coacervation properties in the same way as tropoelastin. Based on these findings, various (FPGVG)_n_ analogues have been developed and investigated for their coacervation abilities^29–33^. We then developed dimer^30,31^ and trimer analogues^33^ of (FPGVG)_n_ and found that oligomerization of (FPGVG)_n_ strengthened the coacervation property, indicating that these oligomerized (FPGVG)_n_ analogues may self-aggregate at low concentrations, that is, in small amounts. The finding that ELPs are temperature-responsive even when the quantity of peptides is low greatly increases their potential for application as biomaterials. As mentioned above, one obvious way to develop short ELP analogues that can be easily synthesized at a low cost is to further shorten the chain length of peptides. It has been reported that the self-assembly ability of ELPs is affected by both the amino acid sequence and the number of hydrophobic amino acids^34^. In other words, an increase in the molecular weight of ELP analogues (i.e., the number of peptide repeats) typically enhances the coacervation ability^35–37^, as does an increase in the concentration of ELPs^35,36,38^. Thus, shortening the chain length can reduce the self-assembly ability of ELPs. On the other hand, removal of hydrophilic residues (such as Gly) from the repeating sequence of ELPs increases the hydrophobicity of the peptide and can increase its self-assembling ability, since it has been revealed that hydrophobic residues tend to increase the coacervation activity of ELPs, while hydrophilic residues decrease it^36,39^. It is also important to note that the deletion of repeat-forming residues alters the secondary structure of ELPs and affects their self-assembly ability^23,39–44^.

In the present study, truncated ELP analogues were designed based on the following concept: (1) Phe residues were retained to maintain hydrophobicity; (2) the sequential amino acid residues, Pro-Gly, on the repetitive sequences were retained as much as possible because they are necessary for the formation of the β-turn structure, which is thought to be important for ELP analogues to undergo coacervation^23,28,39–44^. Based on these concepts, several truncated ELP analogues were synthesized by deleting Gly and Val residues from the (FPGVG)_n_ repetitive sequence, and their self-assembling properties were evaluated to investigate the relationship between thermoresponsiveness and amino acid sequences. To analyze the effect of amino acid sequence changes on the temperature-dependent secondary structure changes of peptides, the secondary structures of truncated ELP analogues were studied using circular dichroism (CD) spectroscopy. Furthermore, microscopy studies of the truncated ELPs were carried out to examine the morphology of the ELP aggregates. In addition, to assess their potential usefulness it is important to develop a functional biomaterial using the newly discovered truncated ELP analogues. In a previous study on the application of ELPs as biomaterials, we reported an ELP analogue, AADAAC-(FPGVG)_4_, which showed thermosensitive coacervation and Cd^2+^ and Zn^2+^ binding activities. In other words, we found that AADAAC-(FPGVG)_4_, which was composed of 26 amino acid residues, exhibited coacervation and metal-binding potency^45^. From an economical point of view, the introduction of this metal-binding sequence into a further shortened ELP analogue would be worthwhile as it would enable the development of a novel environmentally friendly metal-scavenging agent with a shorter amino acid sequence. Thus, in this study, we designed a shortened AADAAC-(FPGVG)_4_-like metal-scavenging agent using a newly discovered truncated ELP analogue and examined its potential usefulness.

## Results and discussion

### Peptide synthesis and purification

**Figure 1:**
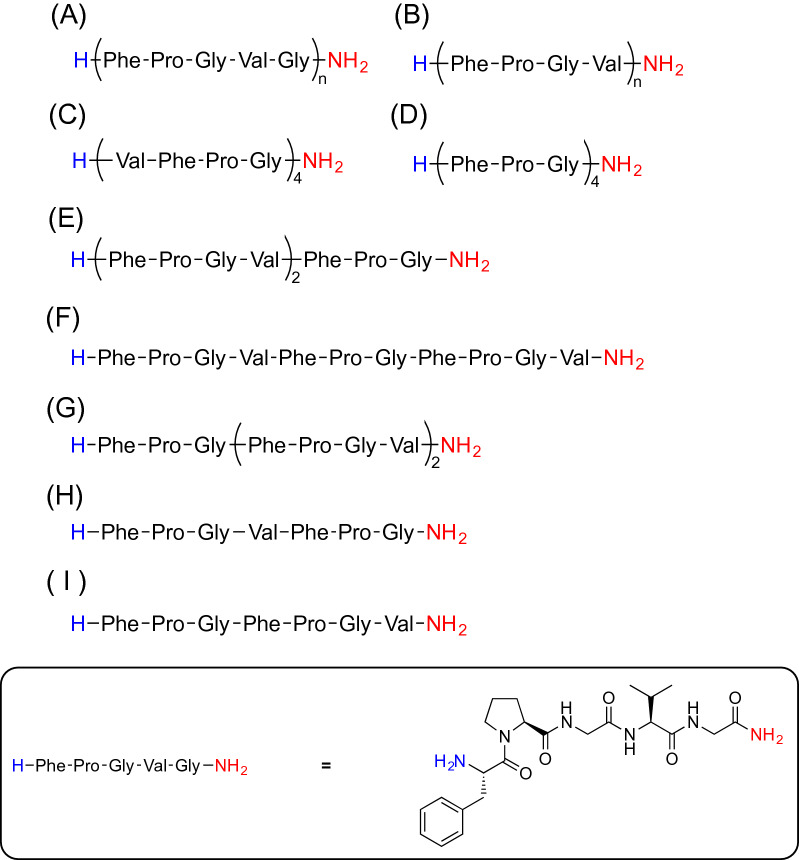
Chemical structures of the elastin-like peptide (ELP) analogues synthesized in this study. The chemical structures of (**A**) (FPGVG)_n_ (n = 4, 5), (**B**) (FPGV)_n_ (n = 2–5), (**C**) (VFPG)_4_, (**D**) (FPG)_4_, (**E**) (FPGV)(FPGV)(FPG), (**F**) (FPGV)(FPG)(FPGV), (**G**) (FPG)(FPGV)(FPGV), (**H**) (FPGV)(FPG), and (**I**) (FPG)(FPGV). The C-terminus of each peptide was capped with an amide group (represented by red letters).

As shown in Fig. 1, we used ELP analogues (FPGVG)_4_ and (FPGVG)_5_ as standard peptides and synthesized 11 truncated ELP analogues as follows: (FPGV)_5_, (FPGV)_4_, (FPGV)_3_, (FPGV)_2_, (VFPG)_4_, (FPG)_4_, (FPGV)(FPGV)(FPG), (FPGV)(FPG)(FPGV), (FPG)(FPGV)(FPGV), (FPGV)(FPG). and (FPG)(FPGV). Notably, the amino acid composition of (VFPG)_n_ was the same as that of (FPGV)_n_, but the amino acid sequence was shifted by one residue. Peptide synthesis was carried out using the conventional solid-phase peptide synthesis procedure (see supplementary information). The synthesized peptides were purified using RP-HPLC. The purity and molecular weight of each peptide were confirmed using RP-UPLC-MS (Table S1 and Fig. S1). Molecular weights of the peptides were also confirmed by MALDI-TOF-MS (MALDI-8200, Shimadzu Co. Kyoto, Japan) (Table S1 and Fig. S2) The results indicated that the peptide analogues were successfully obtained with high purity.

### Turbidity measurement of the truncated ELP analogues

**Figure 2:**
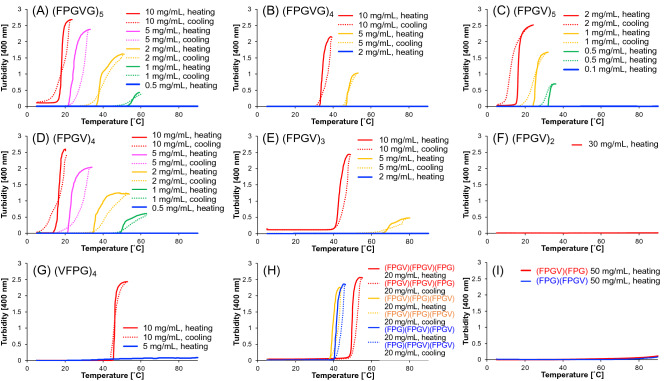
Turbidity profiles of the short elastin-like peptide (ELP) analogues consisting of combinations of FPG and FPGV sequences. Turbidity changes of the synthesized peptides in phosphate buffer (27.4 mM Na_2_HPO_4_, 17.8 mM NaH_2_PO_4_, pH 7.4) associated with heating (solid lines) and cooling (dashed lines). (**A**) (FPGVG)_5_, (**B**) (FPGVG)_4_, (**C**) (FPGV)_5_, (**D**) (FPGV)_4_, (**E**) (FPGV)_3_, (**F**) (FPGV)_2_, (**G**) (VFPG)_4_, (**H**) (FPGV)(FPGV)(FPG) (red lines), (FPGV)(FPG)(FPGV) (orange lines), (FPG)(FPGV)(FPGV) (blue lines), and (**I**) (FPGV)(FPG) (red line), (FPG)(FPGV) (blue line).

Turbidity measurements were conducted to examine the coacervation ability of (FPGV)_n_ in phosphate buffer solution (pH 7.4). To quantitatively evaluate the coacervation properties of the synthesized analogues, the transition temperature (*T*_t_) was calculated from the turbidity change associated with the temperature increase (Fig. 2). The concentration of each peptide in the phosphate buffer was adjusted according to the solubility of the peptide. Turbidity measurements of standard ELP analogues ((FPGVG)_5_ and (FPGVG)_4_) were performed (Table 1 and Fig. 2A,B). Under the experimental conditions in this study, the *T*_t_ of (FPGVG)_5_ and (FPGVG)_4_ were 20.5 ℃ and 33.8 ℃ at a concentration of 10 mg/mL, respectively. The *T*_t_ of these peptides increased with decreasing peptide concentration (Fig. 2A,B), in accordance with previous report^35,37^. The coacervation ability of (FPGV)_n_ (n = 5–2) was also evaluated. Even though the chain length of (FPGV)_n_ was shorter than that of (FPGVG)_n_, the *T*_t_ of (FPGV)_n_ was lower than that of (FPGVG)_n_ at the same repetition number (n = 5 or 4) (Table 1 and Fig. 2C,D ). For example, the *T*_t_ of (FPGV)_5_ was 17.9 ℃ at a concentration of 2.0 mg/mL. The relationship between the *T*_t_ of ELPs and peptide concentration was further investigated for ELPs that exhibited strong self-assembling ability, namely ((FPGVG)_5_, (FPGV)_5_, and (FPGV)_4_), based on the equation *T*_t_ = *a* ln(C) + *b* (where C is the molar concentration of the peptide) given by Meyer and Chilkoti^35^. As a result, the *T*_t_ values of these peptides fit well with the equation. (Fig. S3). Interestingly, (FPGVG)_5_ and (FPGV)_4_ showed similar profiles in this relationship, indicating that (FPGV)_4_ could be compatible with (FPGVG)_5_ as thermoresponsive material. Reversible coacervation properties were also observed in the (FPGV)_3_ profile (Table 1 and Fig. 2E). This result indicated that (FPGV)_n_ possessed a higher coacervation ability than (FPGVG)_n_. In addition, it was also revealed that the fifth Gly residue on the repetitive sequence of (FPGVG)_5_ was not necessarily required for the reversible self-assembly of ELPs. The self-assembly ability of (FPGV)_n_ was reduced by decreasing the number of repetitive sequences; (FPGV)_2_ did not exhibit turbidity change even at a concentration of 30 mg/mL (Table 1 and Fig. 2F). Furthermore, we evaluated the coacervation ability of an analogue (VFPG)_4_, with a shifted amino acid sequence of (FPGV)_4_ (Table 1 and Fig. 2G) and found that the coacervation activity of (VFPG)_4_ was weaker than that of (FPGV)_4_, although these two peptides have the same amino acid chain length and composition. The results of UPLC analysis suggested that this difference was due to the hydrophobicity of the peptide in solution; (FPGV)_4_ was more hydrophobic than (VFPG)_4_ (UPLC retention time was 2.696 min for (FPGV)_4_ and 2.493 min for (VFPG)_4_). Comparing the sequences of (FPGV)_4_ and (VFPG)_4_, there was a difference in the number of Phe-Pro-Gly-Val (of Xxx_1_-Pro-Gly-Xxx_2_) components required to form the β-turn structure. That is, (FPGV)_4_ has four components, whereas (VFPG)_4_ possesses only three of these components due to the shifting of the amino acid sequence. Thus, it was considered that the number of Phe-Pro-Gly-Val components could significantly affect the hydrophobicity and secondary structure of ELP analogues in coacervation.

**Table 1:** The phase transition temperature (*T*_t_) values of truncated ELP analogues.

Peptide	Concentration		*T*_t_ ( ℃)
	mg/mL	mM	
(FPGVG)_5_	10	4.34	20.5 ± 0.1
	5.0	2.17	25.6 ± 1.2
	2.0	0.87	39.0 ± 0.2
	1.0	0.43	56.6 ± 1.3
	0.5	0.21	Not determined
(FPGVG)_4_	10	5.42	33.8 ± 0.3
	5.0	2.71	47.5 ± 0.3
	2.0	1.08	Not determined
(FPGV)_5_	2.0	0.99	17.9 ± 0.7
	1.0	0.50	24.9 ± 0.3
	0.5	0.25	33.3 ± 0.2
	0.1	0.05	Not determined
(FPGV)_4_	10	6.18	17.0 ± 0.2
	5.0	3.09	23.4 ± 0.1
	2.0	1.24	36.7 ± 0.3
	1.0	0.62	51.3 ± 0.1
	0.5	0.31	Not determined
(FPGV)_3_	10	8.21	45.2 ± 0.9
	5.0	4.10	71.6 ± 0.8
	2.0	1.64	Not determined
(FPGV)_2_	30	36.7	Not determined
(VFPG)_4_	10	6.18	45.3 ± 0.6
	5.0	3.09	Not determined
(FPG)_4_	10	8.18	Not determined^a^
(FPGV) (FPGV) (FPG)	20	17.9	50.3 ± 0.9
(FPGV) (FPG) (FPGV)	20	17.9	40.5 ± 0.5
(FPG) (FPGV) (FPGV)	20	17.9	42.3 ± 0.6
(FPGV) (FPG)	50	69.5	Not determined
(FPG) (FPGV)	50	69.5	Not determined

Mean *T*_t_ values with SE were shown in the table. Each peptide was dissolved in phosphate buffer (27.4 mM Na_2_HPO_4_, 17.8 mM NaH_2_PO_4_, pH 7.4). The measurements were repeated at least three times. ^a^Since this peptide analogue showed irreversible aggregation and formed insoluble filamentous aggregates, *T*_t_ value could not be determined.

To examine whether the sequence could be further shortened, (FPG)_4_ was synthesized and its coacervation ability was investigated. However, once the peptide was dissolved in the aqueous solution at 10 mg/mL, (FPG)_4_ formed irreversible aggregates with elapsed time without a temperature rise. Therefore, turbidity measurements could not be conducted for (FPG)_4_. Other truncated ELP analogues consisting of three sequence units, (FPGV)(FPGV)(FPG), (FPGV)(FPG)(FPGV), and (FPG)(FPGV)(FPGV), combined with one FPG sequence and two FPGV sequences, were synthesized to investigate the effects of introducing one FPG sequence, which potentially exhibits irreversible aggregation in (FPGV)_3_. (FPGV)(FPGV)(FPG), (FPGV)(FPG)(FPGV), and (FPG)(FPGV)(FPGV) exhibited coacervation at a concentration of 20 mg/mL, indicating that the concentration of (FPGV)(FPGV)(FPG), (FPGV)(FPG)(FPGV), and (FPG)(FPGV)(FPGV) needed to be doubled to show coacervation at the same temperature as (FPGV)_3_ (Table 1 and Fig. 2H). In comparison with these three analogues, the analogues consisting of one FPG sequence and one FPGV sequence, namely, (FPGV)(FPG) and (FPG)(FPGV), did not show coacervation even at a concentration of 50 mg/mL (Table 1 and Fig. 2I). Hence, these results suggested that the introduction of the FPG sequence did not improve the coacervation ability, but worsened it. In conclusion, these turbidity measurements of the truncated ELP analogues demonstrated that novel truncated ELP analogues, (FPGV)_n_, can be utilized as useful thermoresponsive biomaterials that are easily synthesized.

### Size distribution of the coacervates of the truncated ELP analogues

**Figure 3:**
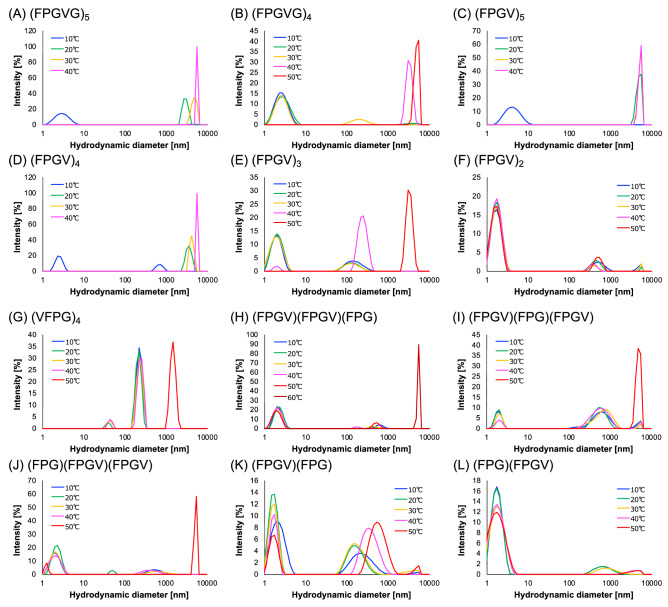
Particle size distribution of the truncated ELP analogues consisting of combinations of FPG and FPGV sequences. The size distribution of the coacervates of the synthesized peptides in phosphate buffer (27.4 mM Na_2_HPO_4_, 17.8 mM NaH_2_PO_4_, pH 7.4) at various temperatures was analyzed via DLS. (**A**) (FPGVG)_5_ (10 mg/mL), (**B**) (FPGVG)_4_ (10 mg/mL), (**C**) (FPGV)_5_ (2.0 mg/mL), (**D**) (FPGV)_4_ (10 mg/mL), (**E**) (FPGV)_3_ (10 mg/mL), (**F**) (FPGV)_2_ (30 mg/mL), (**G**) (VFPG)_4_ (10 mg/mL), (**H**) (FPGV)(FPGV)(FPG) (20 mg/mL), (**I**) (FPGV)(FPG)(FPGV) (20 mg/mL), (**J**) (FPG)(FPGV)(FPGV) (20 mg/mL), (**K**) (FPGV)(FPG) (50 mg/mL), and (**L**) (FPG)(FPGV) (50 mg/mL).

Temperature-responsive behavior of the truncated ELP analogues was investigated via size distribution analyses of the coacervates in each peptide solution with the temperature ranging from 10 to 50 ℃ using dynamic light scattering (DLS) (Fig. 3). The size distribution histograms of ELP analogues were converted from the DLS autocorrelation curves (Fig. S4) by a cumulant fit performed by Zetasizer software. In the DLS of (FPGVG)_5_, the hydrodynamic diameters of the peptides were observed as approximately 3 nm at 10 ℃, whereas it was increased to 3–5 µm at temperatures above 20 ℃ (Fig. 3A). Thus, it was indicated that micrometer-sized aggregates were formed at temperatures higher than *T*_t_. When the solution temperature was raised to 50 ℃, the particle size could not be measured, probably due to the precipitation of the particles. Subsequently, the size distributions in other peptide analogue solutions were measured. Owing to the strong self-association ability, (FPGV)_5_ and (FPGV)_4_ rapidly formed micrometer-sized particles at 20 ℃ (Fig. 3C,D). In addition, when the solution temperature of these peptides was above *T*_t_, the tail of the DLS correlogram showed large delay time (= 100,000 µs), suggesting that the particles of these peptides matured to large aggregates (Fig. S4A,C, and D). In contrast, (FPGVG)_4_ and (FPGV)_3_, possessing moderate self-association ability, formed sub-micron particles below *T*_t_, which grew into micrometer-sized particles above *T*_t_ (Fig. 3B,E ). This stepwise process was similar to the non-linear ELPs that we previously reported^31–33^. A similar process was observed for (VFPG)_4_, which exhibited moderate self-aggregation ability (Fig. 3G). Furthermore, DLS analyses were performed for the analogues combined with one FPG sequence and two FPGV sequences, namely, (FPGV)(FPGV)(FPG), (FPGV)(FPG)(FPGV), and (FPG)(FPGV)(FPGV). Among these, the self-association ability of (FPGV)(FPGV)(FPG) was weaker than that of the other two; the particle size of (FPGV)(FPGV)(FPG) increased to micrometer-order only at 60 ℃ (Fig. 3H–J). This could be due to the smaller number of Xxx_1_-Pro-Gly-Xxx_2_ components compared to (FPGV)(FPG)(FPGV) and (FPG)(FPGV)(FPGV). Therefore, it was confirmed that the number of Xxx_1_-Pro-Gly-Xxx_2_ components could significantly affect the self-association ability of ELP analogues. (FPGV)_2_, (FPGV)(FPG), and (FPG)(FPGV) did not show micrometer-sized particles even when the solution temperature was increased to 50 ℃ (Fig. 3F,K,L). In addition, (FPG)_4_ formed irreversible aggregates in the aqueous solution at 10 ℃ and DLS measurements could not be conducted. All these results were consistent with those obtained from turbidity measurements of the peptide analogues. Regarding autocorrelation curves, even the truncated ELP analogues that did not exhibit apparent phase transition in turbidity measurements showed multimodal distribution of particle size at lower temperatures than their *T*_t_. Therefore, it was considered that the ELPs essentially have self-assembly ability and change into particles of various sizes due to their dissociation and aggregation in solution. Notably, despite having fewer residues than (FPGVG)_5_, (FPGV)_4_ showed almost the same self-assembling ability as (FPGVG)_5_ in the DLS analysis, as well as turbidity analysis. Thus, (FPGV)_4_ was considered to be useful as a thermoresponsive biomaterial with shortened chain length.

### Structural study of the truncated ELP analogues

**Figure 4:**
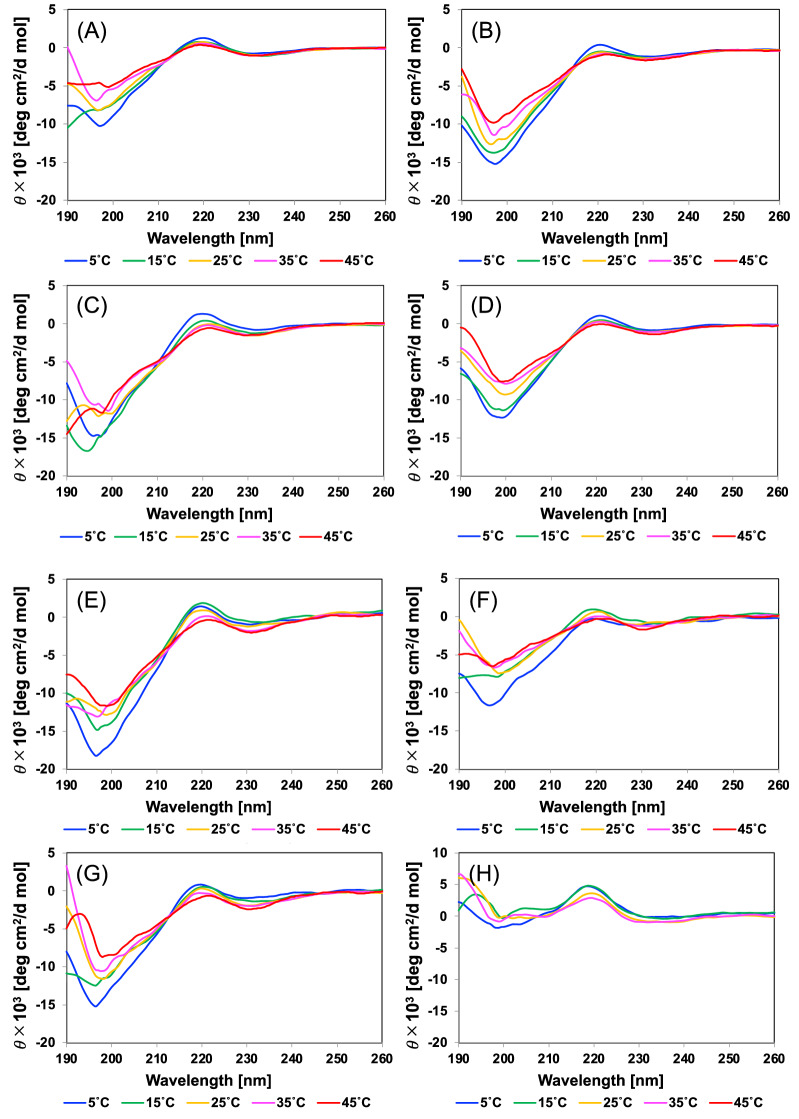
Circular dichroism (CD) spectra of the truncated elastin-like peptide (ELP) analogues. The CD spectra of the truncated ELP analogues are shown. (**A**) (FPGVG)_4_, (**B**) (FPGV)_4_, (**C**) (FPGV)_3_, (**D**) (VFPG)_4_, (**E**) (FPGV)(FPGV)(FPG), (**F**) (FPGV)(FPG)(FPGV), (**G**) (FPG)(FPGV)(FPGV), and (**H**) (FPG)_4_.

To investigate the influence of the sequence alteration on the secondary structure of the truncated ELP analogues, CD spectra measurements of the ELP analogues, (FPGVG)_4_, (FPGV)_4_, (FPGV)_3_, (VFPG)_4_, (FPGV)(FPGV)(FPG), (FPGV)(FPG)(FPGV), (FPG)(FPGV)(FPGV), and (FPG)_4_ were carried out in the range of 260 to 190 nm (Fig. 4). Measurements were performed at peptide concentrations at which these peptides did not exhibit self-assembly to prevent inhibition of CD spectroscopy due to aggregate formation. The spectrum of (FPGVG)_4_ showed the same characteristic bands: a minor negative band at 230 nm, a positive band at 220 nm, and a prominent negative band at 197 nm (Fig. 4A). As the temperature increased, the intensities of these three bands decreased. These characteristic bands were also observed in the CD spectra of F5 in our previous study^29^. In addition, the thermoresponsive truncated ELP analogues, except for (FPG)_4_, also showed almost the same CD spectra profiles (Fig. 4B–G). These temperature-dependent changes in the bands of the truncated ELP analogues were consistent with those of typical polyproline helix II (PPII) structures^46–50^. The spectrum observed in the wavelength range of 230 to 260 nm showed little difference with temperature. In contrast, the shoulder peak at 205 nm emerged with increasing temperature. These results indicated that the proportion of β-turn structures increases with rising temperature^28^. These CD measurement results suggested that the coacervatable truncated ELP analogues formed a PPII-like helical structure at low temperature in solution. Along with the denaturation of the PPII-like helical structure during heating, the characteristics of the β-turn structures emerged. Therefore, this structural change is considered an important feature of the peptide analogues that exhibit coacervation. In contrast, the spectrum of (FPG)_4_, which exhibited irreversible aggregation, was different from that of the other truncated ELP analogues. The spectrum showed characteristic bands: a minor negative band at 230 nm, a prominent positive band at 220 nm, and a negative band at 200 nm (Fig. 4H). Hence, the strength of their bands was significantly different from that of the others. In addition, an increase in the peak at 205 nm, indicating the formation of a β-turn structure, was not observed with increasing temperature. These results indicate that the deletion of Val-Gly residues in the repetitive sequence altered the characteristics of the secondary structure and aggregation properties of the ELP analogues.

**Figure 5:**
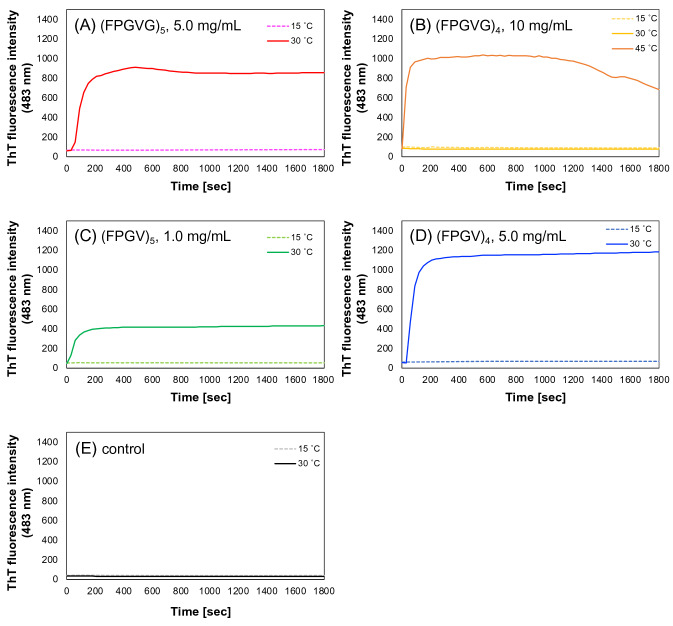
Time-lapse ThT fluorescence assay of truncated ELP analogues. Time-lapse ThT fluorescence assay monitoring below and above the *T*_t_. (**A**) (FPGVG)_5_ (5 mg/mL), (**B**) (FPGVG)_4_ (10 mg/mL), (**C**) (FPGV)_5_ (1 mg/mL), (**D**) (FPGV)_4_ (5 mg/mL), and (**E**) control (no peptide included). Each peptide was dissolved in phosphate buffer (27.4 mM Na_2_HPO_4_, 17.8 mM NaH_2_PO_4_, pH 7.4) and ThT was added at a final concentration of 50 µM.

To further investigate the conformational changes involved in peptide self-assembly, thiofalvin T (ThT) fluorescence measurements were performed (Fig. 5). In each peptide solution below *T*_t_, the fluorescent intensity of ThT in each peptide solution remained low below the *T*_t_ of each peptide, whereas it immediately increased and reached a plateau after 5 min above *T*_t_. (Fig. 5A–D). The fluorescent intensity of ThT in 10 mg/mL of (FPGVG)_4_ solution at 30 ℃ was nearly identical to that at 15 ℃, whereas it significantly elevated at 45 ℃ (Fig. 5B). Furthermore, in the absence of ELP analogues, fluorescent intensity was not changed by temperature rise (Fig. 5E). This result suggests that the truncated ELP analogues changed to β-sheet-rich structures during self-assembly. We have previously reported that dimeric peptides of (FPGVG)_5_ formed sheet-turn-sheet motifs, which are considered to be important for peptide accumulation and aggregation^30^. In addition, the turn structure formed by the Phe-Pro-Gly-Val sequence in (FPGVG)_5_ is similar to a type-II β-turn structure. Therefore, we concluded that the repetitive sequences of (FPGVG)_n_ and its truncated analogues changed to structures rich in β-sheets and β-turns, which are thought to be important for the self-assembly.

### Morphology of the peptide aggregates

**Figure 6:**
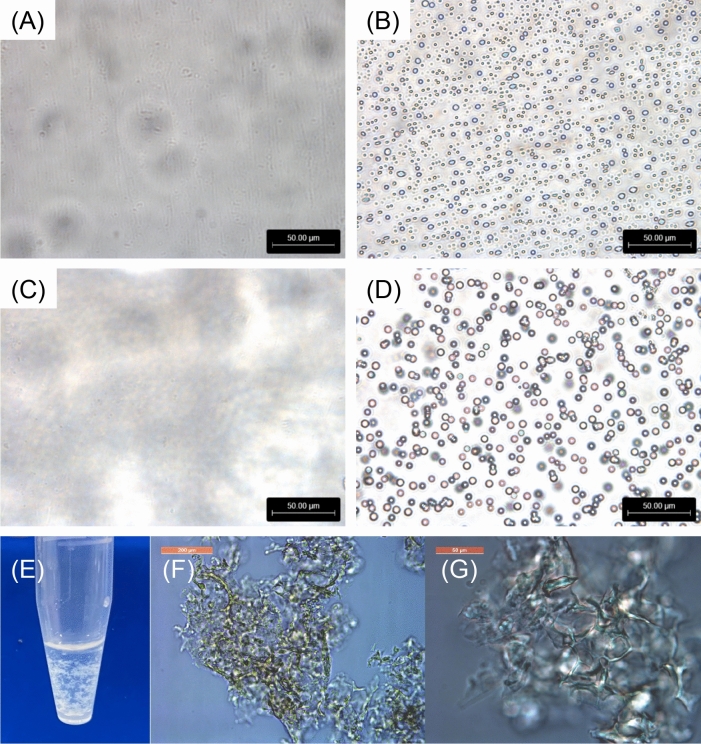
Morphological studies of the truncated elastin-like peptide (ELP) analogues. Optical microscopy images of the truncated ELPs dissolved in phosphate buffer (27.4 mM Na_2_HPO_4_, 17.8 mM NaH_2_PO_4_, pH 7.4) at a concentration of 10 mg/mL. Microscopy images of (**A**) (FPGVG)_4_ (5 °C, × 40), (**B**) (FPGVG)_4_ (40 °C, × 40), (**C**) (FPGV)_4_ (5 °C, × 40), and (**D**) (FPGV)_4_ (25 °C, × 40). (**E**) Image of (FPG)_4_ irreversible aggregates formed in a phosphate buffer solution. Microscopy images of (**F**) (FPG)_4_ (× 10) and (**G**) (FPG)_4_ (× 40) are also shown. Scale bars: 50 μm in (**A**)–(**D**) and (**G**), and 200 μm in (**F**).

**Figure 7:**
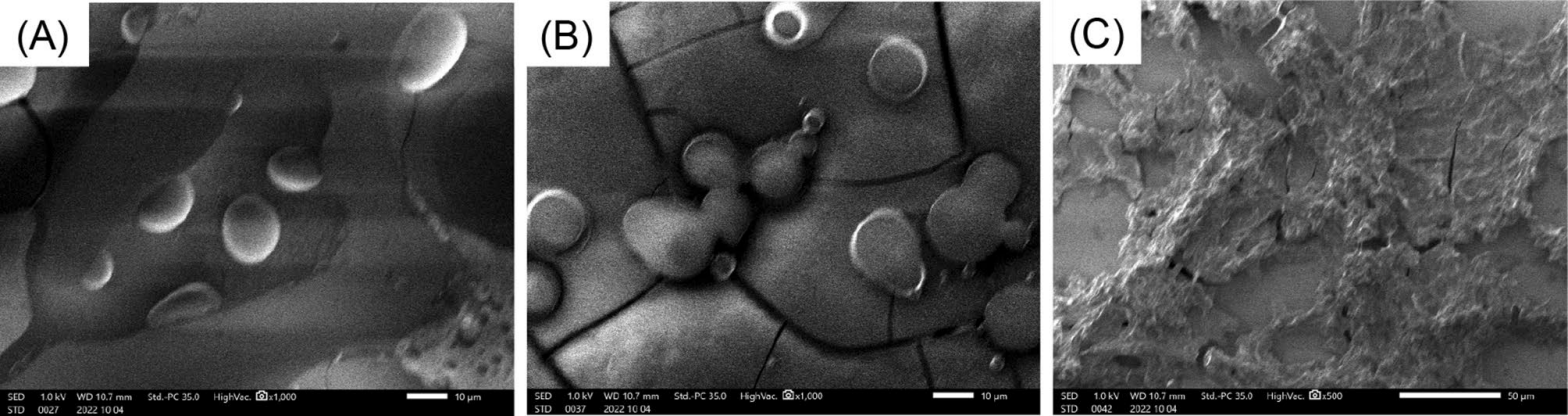
SEM image of self-assembled structures of truncated ELP analogues. SEM images of the self-assembled structures of (**A**) (FPGVG)_4_, (**B**) (FPGV)_4_, and (**C**) (FPG)_4_. The magnifications for the images are 1000 × for (**A**) and (**B**), and 500 × for (**C**).

To investigate the morphological properties of the synthesized peptide analogues, we observed the phosphate buffer solutions containing (FPGVG)_4_, (FPGV)_4_, and (FPG)_4_ at a concentration of 10 mg/mL below or above *T*_t_ using optical microscopy (Fig. 6). Although the (FPGVG)_4_ solution was homogeneous at 5 ℃, (FPGVG)_4_ formed spherical aggregates above *T*_t_ (33.8 ℃ at 10 mg/mL), similar to those of (FPGVG)_5_ (Fig. S5). (FPGV)_4_ also formed spherical aggregates at 25 ℃ (Fig. 6C,D ). As a result of image analyses, (FPGVG)_4_ was mostly distributed as particles of about 1 µm, whereas (FPGV)_4_ showed an increasing distribution of larger (2–4 µm) particles (Figs. S6 and S7). In continuous microscopic observation, the aggregates of (FPGV)_4_ appeared to fuse faster than those of (FPGVG)_4_. This phenomenon was thought to be because (FPGV)_4_, which is more hydrophobic than (FPGVG)_4_, becomes more stable as the surface area of the aggregate decreases. (FPG)_4_, which exhibited irreversible self-assembly, showed aggregates comparable with those of reversible ELPs. As described above, (FPG)_4_ formed irreversible aggregates and did not dissolve again, although this peptide was dissolved in the phosphate buffer solution at a concentration of 10 mg/ml and low temperature (ca. 4 ℃). As shown in Fig. 6E, (FPG)_4_ showed filamentous aggregates and precipitated over time. The shape of the aggregates was amorphous, and their sizes were significantly larger than those of the other aggregates (Fig. 6F,G). This indicated that the irreversible aggregation of (FPG)_4_ is different from the reversible coacervation of ELP analogues in morphology. Further morphological analysis was performed by using scanning electron microscopy (SEM) to obtain structural information of the coacervates of truncated ELP analogues (Fig. 7). In the sample prepared from the solution of (FPGVG)_4_, spherical particles with a diameter of approximately 5–20 μm were observed, which were larger than that observed by optical microscopy (Fig. 7A). This may be due to the fusion of multiple particles during preparation of the sample for SEM, formation larger particles. SEM observation of (FPGV)_4_ showed that multiple spherical particles were fused together (Fig. 7B). On the contrary, the sample of (FPG)_4_ formed amorphous structures and no spherical particles were observed (Fig. 7C). Similar to the above, irreversible aggregates of (FPG)_4_ were hard to solve in water during sample preparation. This result indicated that the reversibility of aggregate formation was lost by the deletion of the Val-Gly residues from the parent repetitive sequence, FPGVG, presumably due to changes in the aggregate structure.

### The properties of AADAAC-(FPGV)_4_

**Figure 8:**
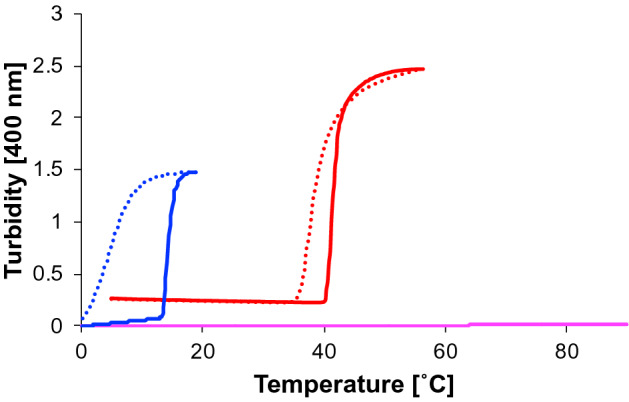
Turbidity profiles of AADAAC-(FPGV)_4_. Turbidity changes of AADAAC-(FPGV)_4_ in Tris–HCl buffer solution (50.0 mM Tris, pH 8.4) associated with heating (solid lines) and cooling (dashed lines). Red lines: 10 mg/mL (4.71 mM) of AADAAC-(FPGV)_4_; magenta lines: 0.5 mg/mL (0.236 mM) of AADAAC-(FPGV)_4_ in the presence of 154 mmol NaCl; blue lines: 0.5 mg/mL (0.236 mM) of AADAAC-(FPGV)_4_ in the presence of 154 mmol NaCl and 0.236 mM CdCl_2_.

From the above-mentioned results regarding the new truncated ELP analogues, it was noted that the FPGV sequence exhibited stronger coacervation ability than the FPGVG sequence. Therefore, to investigate the potential usefulness of the newly developed shortened-chain ELP analogues as biomolecular materials, we developed a new metal-scavenging agent using (FPGV)_n_ by introducing a metal-binding peptide, Ala-Ala-Asp-Ala-Ala-Cys (AADAAC) possessing an affinity for heavy metals such as Cd^2+^ or Zn^51^. This hexapeptide sequence strongly and selectively binds to cadmium above pH 6.0, whereas this peptide releases metal ions below pH 4.0^51^. Recently, we reported that an ELP analogue, AADAAC-(FPGVG)_4_, which was prepared by conjugating an AADAAC sequence at the *N*-terminus of (FPGVG)_4_, can be utilized as a metal scavenger exhibiting high affinity for cadmium ions^45^. In the present study, AADAAC-(FPGV)_4_ was prepared using (FPGV)_4_, which shows stronger coacervation activity than F4 and investigated its properties as a shorter and easy-to-synthesize metal scavenging agent (Table S1 and Fig. S8). To assess the self-assembly property, turbidity measurements of AADAAC-(FPGV)_4_ were carried out under the same conditions as described above (Table 2 and Fig. 8). First, AADAAC-(FPGV)_4_ was dissolved to a concentration of 10 mg/mL in Tris–HCl buffer (pH 8.4) instead of phosphate buffer (pH 7.4), considering its use as a metal scavenging agent under basic conditions. Under these conditions, the *T*_t_ of AADAAC-(FPGV)_4_ was 41.6 ± 1.0 ℃ at a peptide concentration of 10 mg/mL. Since *T*_t_ tended to increase with the proportion of hydrophilic residues in the peptide, the self-assembling ability of AADAAC-(FPGV)_4_ was weakened compared with that of (FPGV)_4_, owing to the presence of hydrophilic Asp and Cys residues in the AADAAC sequence conjugated to (FPGV)_4_^34^. On the other hand, in the presence of Cd^2+^, AADAAC-(FPGV)_4_ was insoluble in the solution. Therefore, the binding of the AADAAC sequence to Cd^2+^ ions was thought to contribute to the enhanced self-assembly of AADAAC-(FPGV)_4_ in the presence of the metal ions. In other words, this result suggests that the self-assembly ability of this peptide was enhanced by the presence of Cd^2+^. This characteristic of AADAAC-(FPGV)_4_ was similar to that of AADAAC-(FPGVG)_4_, which we previously reported^45^. Subsequently, to verify whether this peptide can be used as a metal recovery agent at a lower peptide concentration, turbidity measurement using a low-concentration peptide solution (0.5 mg/mL, 0.236 mM) was performed. At this peptide concentration, it was considered that the peptide solution did not exhibit coacervation, since the self-association ability of an ELP depends on its concentration^35,36,38^. Thus, turbidity was measured in Tris–HCl buffer (50.0 mM Tris, 0.236 mM CdCl_2_, pH 8.4) containing 0.9% NaCl (154 mM, equivalent to saline), because it has been reported that salts such as NaCl increase the self-association and decrease the *T*_t_ of ELPs^52^. As a result, the *T*_t_ of AADAAC-(FPGV)_4_ was 13.8 ± 0.1 ℃ at a peptide concentration of 0.5 mg/mL in the presence of an equimolar amount of Cd^2+^, whereas the peptide solution did not show coacervation in the absence of Cd^2+^. Therefore, this peptide was highly water-soluble in the absence of Cd^2+^ and exhibited high agglutination capacity only in the presence of cadmium ions. Owing to this drastic change in water solubility in the presence and absence of cadmium ions, AADAAC-(FPGV)_4_ was considered to be useful as a Cd^2+^-selective metal-scavenging agent.

**Table 2:** *T*_t_ values of AADAAC-(FPGV)_4_.

Peptide	Concentration of peptide		Additives	*T*_t_ (℃)
	mg/mL	mM		
AADAAC-(FPGV)_4_	10	4.71	–	41.6 ± 1.0
	10	4.71	4.71 mM CdCl_2_	Not dissolved
	0.5	0.236	154 mM NaCl, 0.236 mM CdCl_2_	13.8 ± 0.1
	0.5	0.236	154 mM NaCl	Not determined

Mean *T*_t_ values with SE were shown in the table. The peptide was dissolved in Tris–HCl buffer solution (50.0 mM Tris, pH 8.4). The measurements were repeated at least three times.

**Figure 9:**
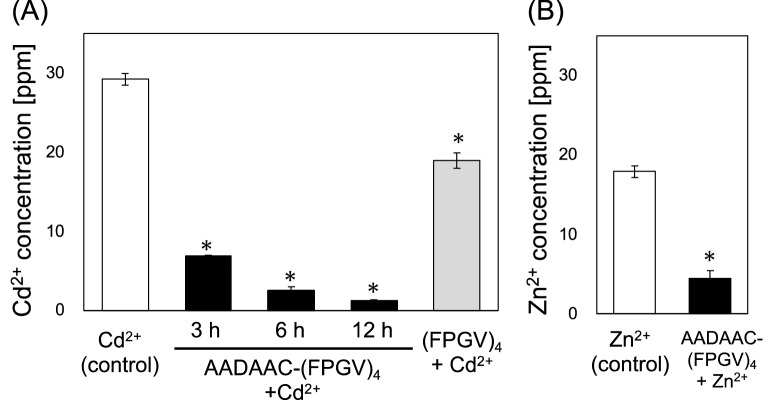
Metal binding affinity of AADAAC-(FPGV)_4_. (**A**) The concentration of Cd^2+^ in the supernatant of CdCl_2_ solution (0.236 mM) was measured 3 h, 6 h, and 12 h after treatment with AADAAC-(FPGV)_4_ at a concentration of 1.0 mg/mL (black bars). Cd^2+^ concentration in the solution 12 h after treatment with the same concentration of (FPGV)_4_ was also shown (gray bar). (**B**) The concentration of Zn^2+^ in the supernatant of ZnCl_2_ solution (0.236 mM) was measured 12 h after treatment with AADAAC-(FPGV)_4_ at a concentration of 1.0 mg/mL. The molar concentration ratio of AADAAC-(FPGV)_4_ and metal ion was 2:1. *P < 0.01, concentration after treatment with the peptide compared to that of the control (*t*-test).

Subsequently, the metal-binding ability of AADAAC-(FPGV)_4_ was assessed using colorimetric analysis using an aqueous solution of CdCl_2_ or ZnCl_2_ (Table 3 and Fig. 9). The interaction of AADAAC sequences with metal ions has been investigated in previous studies by Nihi et al*.* and has been shown to have multiple pH-dependent interactions^51^. In addition, as revealed in our previous work, multiple AADAAC-(FPGVG)_4_ molecules coordinated with one Cd^2+^ and Zn^2+^ ion^45^. Therefore, an excess amount (twofold) of AADAAC-(FPGV)_4_ with respect to metal ions was used as a metal scavenging agent. As a result, Cd^2+^ concentration was significantly reduced by treatment with AADAAC-(FPGV)_4_. The removal efficacy of Cd^2+^ was improved by elongation of the incubation time at 4 ℃ before phase separation; Cd^2+^ concentration in the supernatant was reduced to 4.9% by incubation for 12 h before heating (Fig. 9A). On the other hand, when the Cd^2+^ solution was treated with (FPGV)_4_ without the AADAAC sequence, the Cd^2+^ removal efficiency was only 27.2% even after 12 h incubation. This partial reduction in the Cd^2+^ concentration could be attributed to metal adsorption on the (FPGV)_4_ peptides themselves during coacervate formation. Thus, the high removal efficiency of Cd^2+^ by AADAAC-(FPGV)_4_ was inferred to be due to metal adsorption by the AADAAC sequence. Similar measurements were also performed for Zn^2+^ using ZnCl_2_ solution for AADAAC-(FPGV)_4_. As shown in Fig. 9B, although the Zn^2+^ concentration in the supernatant was also decreased by treatment with this peptide, the removal efficiency was lower than that of Cd^2+^. This result was similar to our previous study related to AADAAC-(FPGVG)_4_^45^. Our previous study implied that most of the removed metal ions were incorporated into the aggregates formed by the peptides, rather than captured by AADAAC sequence. It was considered that the binding of the metal ions to AADAAC sequence significantly increased the hydrophobicity of AADAAC-containing ELPs and induced self-assembly. The higher removal efficiency of Cd^2+^ compared to that of Zn^2+^ could be attributed to Cd^2+^ forming a more thermodynamically stable complex with the AADAAC sequences, promoting the self-assembly of the ELPs. In summary, using the truncated ELP, (FPGV)_4_, synthesized in this study, we were able to fabricate a peptide analogue with a metal recovery capacity. In addition to increasing the hydrophobicity of ELPs, it is possible to develop even shorter ELP analogues. This was achieved by elucidating the aggregation mechanism of truncated ELP analogues and modifying them to further increase aggregation based on the observed results, such as the deletion of the Gly residue found in this study.

**Table 3:** The removal efficiency of metal ions with the AADAAC-(FPGV)_4_ treatment.

Peptide	Incubation time	Metal ion	Concentration of metal ion in the supernatant	Removal efficiency
	(h)		(ppm)	(%)
- (control)	12	Cd^2+^	26.1 ± 1.80	–
AADAAC-(FPGV)_4_, 1.0 mg/mL	3	Cd^2+^	6.8 ± 0.11	78.8 ± 0.003
	6	Cd^2+^	2.5 ± 0.49	90.9 ± 0.018
	12	Cd^2+^	1.3 ± 0.046	95.1 ± 0.002
(FPGV)_4_, 1.0 mg/mL	12	Cd^2+^	19.0 ± 0.97	27.2 ± 0.04
- (control)	12	Zn^2+^	17.9 ± 0.59	–
AADAAC-(FPGV)_4_, 1.0 mg/mL	12	Zn^2+^	4.4 ± 0.008	75.3 ± 0.001

Mean removal efficiency with SE were shown in the table. Each peptide was dissolved in Tris–HCl buffer solution (50.0 mM Tris, 599 mM NaCl, pH 8.0). The measurements were repeated at least three times. The molar concentration ratio of AADAAC-(FPGV)_4_ and metal ion was 2:1 (0.476 mM : 0.236 mM).

## Conclusions

In this study, novel ELP analogues were synthesized to develop shortened functional peptides with coacervation properties. These ELP analogues are composed of proteinogenic natural amino acids and are expected to be used as biomaterials with a small environmental impact. In general, ELPs are biopolymers that are typically composed of many amino acid residues that are expressed in a variety of protein expression systems. Among such ELPs, (VPGVG)_n_ (n > 40) is widely used in the development of biomaterials, although it is extremely difficult to synthesize chemically. In contrast, the newly developed (FPGV)_4_ and other truncated ELPs based on the (FPGVG)_n_ sequence, which show stronger coacervation activity than (VPGVG)_n_, are relatively easy to synthesize chemically. Despite a shortened chain length consisting of only 16 natural amino acid residues without any other components, (FPGV)_4_ exhibited reversible temperature-dependent coacervation features and formed droplets to precipitate coacervates. The discovery of (FPGV)_4_ will contribute to the possibility of mass production of functional peptides and ELPs using chemical industrial methods more easily and inexpensively than before.

Today's growing concern for a sustainable society calls for the conversion of raw materials for industrial products from synthetics derived from petrochemical technology to natural products, especially inexpensive and non-toxic compounds. Peptides and proteins can perform a variety of functions by combining diverse amino acids, and even today, the search for these new functions is underway. Since the development of Merrifield's solid-phase method, various condensation agents and resins for solid-phase synthesis have been developed, and it is now possible to prepare peptides rather easily. However, since the mass synthesis of peptides is still a costly and difficult task, a shorter peptide chain length and repeat sequence will simplify the mass synthesis of peptides and contribute to reducing the problems associated with peptide synthesis. Therefore, it is necessary to improve the synthesis method of AADAAC-(FPGV)_4_, which was developed in this study, and to establish a procedure to prepare this peptide more easily and in large quantities. Future challenges include the introduction of target recognition signal sequences for additional functions, confirmation of stability in the operating environment, and the establishment of a mass synthesis method using the liquid phase method. Once an inexpensive method for mass preparation is established, the usefulness of peptides will increase further, and the search for new functions of peptides, which are small molecules compared to proteins, will become more important.

## Materials and methods

### Chemicals

Peptide synthesis was carried out using a conventional Fmoc-strategy with Fmoc-amino acids (Merck Ltd. Darmstadt, Germany) and Fmoc-NH-SAL-MBHA resin (100–200 mesh, Watanabe Chemical Industries Ltd. Hiroshima, Japan). Other reagents used for peptide synthesis, namely, *N,N*-diisopropylethylamine and trifluoroacetic acid, were purchased from Watanabe Chemical Industries Ltd., and 2-(1H-benzotriazole-1-yl)-1,1,3,3-tetramethyl uronium hexafluorophosphate and 1-hydroxybenzotriazole were purchased from Kokusan Chemical Co., Ltd. (Tokyo, Japan). Triisopropylsilane and xylenol orange (XO) were purchased from Tokyo Chemical Industry Co., Ltd. (Tokyo, Japan). Hydrochloric acid (HCl), 1,2-ethanedithiol, CdCl_2_⋅2H_2_O, and tris(hydroxymethyl)aminomethane (Tris) were purchased from Nacalai Tesque Co. Ltd. (Kyoto, Japan). ZnCl_2_ was purchased from FUJIFILM Wako Pure Chemical Corporation (Osaka, Japan). Water for the experiments was purified using a Milli-Q Integral 3 system (Merck Millipore, Darmstadt, Germany). Other solvents and reagents were obtained from commercial suppliers and used without further purification.

### Synthesis of ELPs and their analogues

Peptide synthesis was performed using the same method as previously reported for H-AADAAC-(FPGVG)_4_-NH_2_, (in there, it was abbreviated as AADAAC-F4)^45^. In principle, the synthetic peptides in this study were prepared with the *N*-terminus free and the C-terminus amide. Briefly, the peptide analogues, (FPGV)_n_ (n = 3, 4, or 5), (VFPG)_4_, (FPG)_4_, (FPGV)(FPGV)(FPG), (FPGV)(FPG)(FPGV), (FPG)(FPGV)(FPGV), (FPGV)(FPG), (FPG)(FPGV), and AADAAC-(FPGV)_4_ were synthesized using an ABI 433A peptide synthesizer (Applied Biosystems, Foster city, CA, USA) and the FastMoc 0.25 mmol program included in the SynthAssist™ 2.0 software (Applied Biosystems) or CSBIO II (Menlo Park, CA). Before final purification using reversed-phase (RP)-HPLC, the synthesized peptide analogues were pre-purified using a Sep-Pak Vac 35 cc C18 cartridge (Waters Co., Milford, MA). Further purification was performed using an RP-HPLC system (The Breeze™ 2 HPLC System, Waters Co.) equipped with a C8 column (COSMOSIL 5C8-AR-300 packed column, 20 mm I. D. × 150 mm, C8-AP 5 μm, 300 Å, Nacalai Tesque Inc.). The details of the peptide synthesis and purification methods are shown in the Supplementary Information file.

The previously synthesized and reported peptide analogues H-(FPGVG)_n_-NH_2_ (n = 4 or 5 (abbreviated as (FPGVG)_4_ and (FPGVG)_5_, respectively) were used in this study^33^.

### Turbidity measurement

Turbidity measurements were performed in accordance with previous reports^33^. The temperature-dependent self-assembling properties of the ELP analogues were evaluated using a JASCO V-660 spectral photometer (JASCO Co., Tokyo, Japan). Peptide sample solutions of different concentrations (2.0, 5.0, 10, 20, 30, or 50 mg/mL) were prepared by dissolving each peptide (except for AADAAC-(FPGV)_n_) in phosphate buffer (27.4 mM Na_2_HPO_4_, 17.8 mM NaH_2_PO_4_, pH 7.4). AADAAC-(FPGV)_4_ was dissolved in Tris–HCl buffer solution (50.0 mM Tris, at pH 8.4 or 50.0 mM Tris, 154 mM NaCl, at pH 8.4) to prepare sample solutions of a concentration of 0.5 or 10 mg/mL in the absence or presence of the equimolar amount of CdCl_2_. Turbidity was measured at 400 nm with increasing or decreasing temperature at a rate of 0.5 ℃/min from 5 ℃. The concentration of each sample solution was measured at least three times. The self-assembling property was described by the phase transition temperature (*T*_t_), which is the temperature at which the turbidity of the solution reaches half the maximum value.

### Dynamic light scattering (DLS) analysis

The distribution of the particle size in the truncated ELP analogue solution was analyzed via DLS using Zetasizernano ZS (Malvern Instruments Ltd., Worcestershire, U.K.) in a measurement cell ZEN0112 (Malvern Instruments Ltd.)^33^. Peptide samples of different concentrations (2.0, 10, 20, 30, or 50 mg/mL) were prepared by dissolving each peptide in phosphate buffer (27.4 mM Na_2_HPO_4_, 17.8 mM NaH_2_PO_4_, pH 7.4) and filtered using the Millex-LG filter (pore size 0.2 µm, Merck Millipore) before measurement. DLS analysis was performed by increasing the temperature at 10 ℃ intervals from 10 to 50 ℃. Measurement duration was selected automatically. Parameter dataset “protein” (dataset: refractive index, 1.450; absorption, 0.001) was used as the material parameter, and parameter dataset “water” (dataset: refractive index, 1.330; viscosity, 0.8872) was chosen as the dispersant parameter. Attenuation was selected automatically. Each concentration was measured at least three times.

### CD measurement

CD measurement was carried out for (FPGVG)_4_, (FPGV)_4_, (FPGV)_3_, (VFPG)_4_, (FPGV)(FPGV)(FPG), (FPGV)(FPG)(FPGV), (FPG)(FPGV)(FPGV), and (FPG)_4_ in a 1.0 mm path-length cuvette using a J-725 spectropolarimeter (JASCO Co.)^33^. Each peptide was dissolved in phosphate buffer (27.4 mM Na_2_HPO_4_, 17.8 mM NaH_2_PO_4_, pH 7.4) to a concentration of 0.1 mg/mL. The spectra of the sample solutions were measured in the range of 190 to 260 nm at cell temperatures between 5 and 45 ℃. To equilibrate the sample temperature, each measurement was performed at least 3 min after the solution reached the target temperature. The spectra of the sample peptides were obtained by subtracting the solvent spectra (without peptide) obtained under the same conditions. Spectral smoothing was performed using Savitzky–Golay smoothing filters.

### Thioflavin T fluorescence assay

Thiofalvin T (ThT) fluorescence measurements were performed for (FPGVG)_5_, (FPGVG)_4_, (FPGV)_5_, and (FPGV)_4_. Each peptide was dissolved in phosphate buffer (27.4 mM Na_2_HPO_4_, 17.8 mM NaH_2_PO_4_, pH 7.4) to different concentrations (1.0 mg/mL for (FPGV)_5_, 5.0 mg/mL for (FPGVG)_5_ and (FPGV)_4_, and 10 mg/mL for (FPGVG)_4_, respectively). Then, 1 mM of ThT aqueous solution was added to each sample at a final ThT concentration of 25 μM) followed by incubation for 1 h at 4 °C. The fluorescence intensity was measured using a FP-8500 spectrofluorometer (JASCO Co.). The excitation was set at 446 nm and the emission was recorded at 483 nm. The fluorescence intensities of samples were monitored at 15 °C for 30 min. Then, the sample temperature was raised to 30 °C and the measurements were performed for another 30 min. The same measurement was also carried out at 45 °C for (FPGV)_4_.

### Microscopy study of the synthesized peptides

The morphology of the coacervates of (FPGVG)_4_, (FPGV)_4_, and (FPG)_4_ was observed using optical microscopy^33^. Light field observation was performed using a Leica DM IL LED microscope (Leica Microsystems CMS, Wetzlar, Germany) equipped with HI PLAN 40 × (Leica Microsystems CMS) and HC PLAN 10 × (Leica Microsystems CMS) objectives. Each peptide was dissolved in phosphate buffer (27.4 mM Na_2_HPO_4_, 17.8 mM NaH_2_PO_4_, at pH 7.4) to a concentration of 10 mg/mL and applied on a glass slide. Sample imaging was performed below and above the *T*_t_ of each peptide using a Thermo Plate TP-CHSQM (Tokai Hit Co., Ltd., Shizuoka, Japan). Sample images above *T*_t_ were taken 3 min after the sample reached the target temperature. The details of the image analysis is shown in the Supplementary Information file.

### Scanning electron microscopy

An aqueous solution of 10 mg/mL of (FPGVG)_4_, (FPGV)_4_, and (FPG)_3_ (in phosphate buffer as described above) was dropped onto a cover glass and left at 40 ℃ for air drying. The prepared samples were osmium-coated with HPC-1SW osmium plasma coater (Vacuum Device Co., Ibaraki, Japan) and examined using a JSM-IT700HR InTouchScope™ (JEOL, Tokyo, Japan) at an operating voltage of 1.0 kV.

### Spectrophotometric determination of the affinity of metal ions to AADAAC- (FPGV)_4_

Colorimetric analyses of cadmium ions (Cd^2+^) were carried out to evaluate the number of metal ions absorbed into the coacervation phase of the peptides by using a spectral photometer (JASCO V-660)^45^. Samples of a concentration of 1.0 mg of (FPGV)_4_ (0.618 mM) or AADAAC-(FPGV)_4_ (0.476 mM) in Tris–HCl (50 mM Tris, 599 mM NaCl, pH8.4) containing 0.236 mM of CdCl_2_ or ZnCl_2_ were prepared. The peptide solutions were incubated at 4 ℃ for 3, 6, or 12 h and then at 60 ℃ for 1.5 h to separate into the lower coacervation phase from the upper equilibrium solution phase. After incubation, the peptide solution was immediately centrifuged at room temperature for 2 min (6,200 rpm) to remove aggregates. An aliquot of 333 µL of supernatant of the equilibrium solution phase was added to 600 µL of acetate buffer solution (pH5.4) including 67 μM of XO and incubated for 10 min. Subsequently, the concentrations of Cd^2+^ or Zn^2+^ were determined by measuring the absorbance at 575 nm or 550 nm, respectively, which corresponds to the absorbance of the metal-XO complex. The amount of metal ions absorbed in the peptide solution was determined by the calibration line, which was prepared in the same manner by using a series of standard CdCl_2_ or ZnCl_2_ aqueous solutions of known concentrations. The homogeneity of variances between the concentration of each metal ion in the supernatant and the control solution was confirmed using the F test. The statistical significance of the difference between the concentration of each metal ion in the supernatant and the control solution was determined using the Student's *t*-test. Results were considered statistically significant at *P* values of ≤ 0.01.

## Supplementary Information

Supplementary Information.

## Data Availability

The datasets used during the current study are available from the corresponding author upon reasonable request.
