# Supplementary material for: Development of truncated elastin-like peptide analogues with improved temperature-response and self-assembling properties
Source: Sci Rep. 2022 Nov 12;12:19414. doi: 10.1038/s41598-022-23940-0 (PMC9653453; doi:10.1038/s41598-022-23940-0)
Supplement: Supplementary file 1 — Supplementary Information. [file 41598_2022_23940_MOESM1_ESM.docx]

**Supplementary information**

**Development of truncated elastin-like peptide analogues with improved temperature-response and self-assembling properties**

AUTHOR NAMES
Shogo Sumiyoshi,^1^ Keitaro Suyama,^2^ Naoki Tanaka,^1^ Takumi Andoh,^1^ Akihiko Nagata,^1^ Keisuke Tomohara,^2^ Suguru Taniguchi,^3,4^ Iori Maeda,^3^ and Takeru Nose^1,2,*^

AUTHOR ADDRESS
^1^Laboratory of Biomolecular Chemistry, Department of Chemistry, Faculty and Graduate School of Science, Kyushu University, Fukuoka 819‑0395, Japan.

^2^Laboratory of Biomolecular Chemistry, Faculty of Arts and Science, Kyushu University, Fukuoka 819‑0395, Japan.

^3^Department of Physics and Information Technology, Kyushu Institute of Technology, Iizuka, Fukuoka 820‑8502, Japan.

^4^Present address: Division of Biomedical Sciences, Fukuoka Dental College, Fukuoka 814‑0193, Japan.

Manuscript Correspondence:

Prof. Takeru Nose

Tel: +81-92-802-6025

Fax: +81-92-802-6025

e-mail: nose@artsci.kyushu-u.ac.jp

**Materials and Methods ........................................................................................(S3–S4)**

Synthesis of Elastin-like peptide analogues.

Purification of the peptides.

Size distribution analysis of coacervates using optical microscopy images of ELP analogues.

**Supporting Tables ......................................................................................................(S5)**

Table S1. ELP analogues synthesized in this study.

**Supporting Figures ............................................................................................(S6–S20)**

Figure S1. UPLC-MS analysis of the synthesized ELP analogues in this study.

Figure S2. MALDI-TOF MS spectra of the synthesized peptide analogues.

Figure S3. Relationship between the *T*_t_ and molar concentration of truncated ELP analogues.

Figure S4. Dynamic light scattering (DLS) autocorrelation curves.

Figure S5. Optical microscopy images of (FPGVG)_5_.

Figure S6. Size distribution analysis of coacervates using optical microscopy images of (FPGVG)_4_.

Figure S7. Size distribution analysis of coacervates using optical microscopy images of (FPGV)_4_.

Figure S8. Chemical structure and UPLC-MS analysis of AADAAC-(FPGV)_4_.

**Synthesis of Elastin-like peptide analogues**

Peptide synthesis was performed using the same method as previously reported.^1^ Briefly, the peptide analogues, H-(FPGV)_n_-NH_2_ (n = 3, 4, or 5), H-(VFPG)_n_-NH_2_ (n = 4), H-(FPG)_4_-NH_2_, H-(FPGV)(FPGV)(FPG)-NH_2_, H-(FPGV)(FPG)(FPGV)-NH_2_, H-(FPG)(FPGV)(FPGV)-NH_2_, H-(FPG)(FPGV)-NH_2_, and H-AADAAC-(FPGV)_4_-NH_2_ (AADAAC-(FPGV)_4_) were synthesized by an ABI 433A peptide synthesizer (Applied Biosystems, Foster city, CA, USA). HBTU (0.45 M) and HOBt (0.45 M) in *N*,*N*-dimethylformamide (DMF) were used as the condensing agents for peptide synthesis in the FastMoc 0.25 mmol program included in SynthAssist^TM^ 2.0 software (Applied Biosystems). Peptides were also synthesized using the CSBioII synthesizer with a manufacture’s standard synthesis program. After peptide chain elongation, the peptides except for cysteine containing analogues were cleaved from the resin with a reagents cocktail containing 95% TFA, 2.5% TIS, and 2.5% H_2_O. AADAAC-(FPGV)_4_ was cleaved from the resin with the reagents cocktail containing 94% TFA, 2.5% EDT, 2.5% H_2_O, and 1.0% TIS. After cleavage of the peptide synthesized from the resin, the resulting mixture was poured into 50 mL of diethyl ether and centrifuged to separate peptide precipitates from the cocktail. The resulting peptide precipitate was resuspended in 50 mL of diethyl ether and the resulting solution was centrifuged to remove contaminants. The peptide analogues H-(FPGVG)_n_-NH_2_ (n = 4 or 5, respectively) were previously synthesized and reported, were used in this experiment.^1^

**Purification of the peptides**

Before final purification by reversed-phase (RP)-HPLC, the synthesized peptide analogues were pre-purified by using a Sep-Pak Vac 35 cc C18 cartridge (Waters Co., Milford, MA).^1^ All peptides synthesized were dissolved in 15% acetonitrile aqueous solution and applied to the Sep-Pak cartridge, and an acetonitrile aqueous solution was subsequently poured into the Sep-Pak cartridge. The eluent solution was fractionated every 50 mL. The concentration of acetonitrile in the eluent was gradually increased to 15%, 30%, 40%, 60%, and 99% to separate the peptides. The elution fractions containing the peptides were identified by detecting the absorption at 230, 265, and 280 nm using a JASCO V-660 spectrophotometer. (JASCO Co., Tokyo, Japan). This fraction was evaporated and followed by lyophilization to obtain the peptide powder. Further purification was performed by RP-HPLC (The Breeze^TM^ 2 HPLC System, Waters Co.) using a C8 column (COSMOSIL 5C8-AR-300 Packed Column, 20 mmI.D. x 150 mm, C8-AP 5 μm, 300 Å, Nacalai Tesque Inc.). A solvent system consisting of 0.1% TFA aqueous solution (v/v, solvent A) and mixture of 80% acetonitrile and 20% solvent A (v/v, solvent B) were used for the gradient elution (with a linear gradient of solvent B 20% to 60% over 40 min and flow rate 3.5 ml/min). Purity and molecular weights of the peptides were confirmed by ACQUITY UPLC H-Class (Waters Co.) equipped with an ACQUITY UPLC BEH C-18 column (100 mm, flow rate 0.6 mL/min) (Waters Co.) at 49°C and the eluting product was detected by UV absorption at 225 nm and a quadrupole mass spectrometer, ACQUITY QDa (Waters Co.). The solvent system for UPLC consisted of 0.1% formic acid aqueous solution (v/v, solvent A) and 0.1% formic acid in acetonitrile (v/v, solvent B), and elution was performed with a linear gradient (34% to 56%) of solvent B over 4.23 min. Molecular weights of the peptides were also confirmed by MALDI-TOF MS using MALDI-8200 (Shimadzu Co., Kyoto, Japan).

**Size distribution analysis of coacervates using optical microscopy images of ELP analogues**

Size distribution of coacervates of ELP analogues formed above the *T*_t_ was analyzed using optical microscopy images of each peptide. Image analysis was performed using Image J/Fiji (NIH, USA), an open-source image analysis software.^2^ Spatial calibration was carried out using an error bar in original images. The images of peptide coacervates were converted to a binary image via the menu command Image > Type > 8-bit. Using "analyze particle" command, the Feret diameters of the particles within the image were measured.

**References**

1. Suyama, K.; Mawatari, M.; Tatsubo, D.; Maeda, I.; Nose, T. Simple regulation of the self-assembling ability by multimerization of elastin-derived peptide (FPGVG)_n_ using nitrilotriacetic acid as a building block. *ACS Omega* **2021**, *6*, 5705–5716.
2. Schindelin, J., Arganda-Carreras, I.; Frise, E.; Kaynig, V.; Longair, M.; Pietzsch, T.; Preibisch, S.; Rueden, C.; Saalfeld, S.; Schmid, B.; Tinevez, J.-Y.; White, D. J.; Hartenstein, V.; Eliceiri, K.; Tomancak, P.; Cardona, A. Fiji: an open-source platform for biological-image analysis. *Nat. Methods.* **2012**, *9*, 676–682.

**Table S1. ELP analogues synthesized in this study.**

| Peptide | Retention time (min) | MS (MATLI-TOF) *m/z* | | |
| --- | --- | --- | --- | --- |
|  |  | Composition formula | Calculated | Found |
| (FPGVG)_5_ | 2.691 | C_115_H_158_N_26_O_25_ | 2305.20 [M+H]^+^  2327.18 [M+Na]^+^ | 2305.36  2327.38 |
| (FPGVG)_4_ | 2.251 | C_92_H_127_N_21_O_20_ | 1847.97 [M+H]^+^ | 1847.05 |
| (FPGV)_5_ | 2.855 | C_105_H_143_N_21_O_20_ | 2020.09 [M+H]^+^  2142.07 [M+Na]^+^ | 2019.87  2141.94 |
| (FPGV)_4_ | 2.431 | C_84_H_115_N_17_O_16_ | 1618.88 [M+H]^+^ | 1618.42 |
| (FPGV)_3_ | 1.739 | C_63_H_87_N_13_O_12_ | 1218.67 [M+H]^+^ | 1218.34 |
| (FPGV)_2_ | 0.583 | C_42_H_59_N_9_O_8_ | 818.46 [M+H]^+^ | 817.82 |
| (VFPG)_4_ | 2.493 | C_84_H_115_N_17_O_16_ | 1618.88 [M+H]^+^ | 1619.99 |
| (FPG)_4_ | 1.835 | C_64_H_79_N_13_O_12_ | 1222.60 [M+H]^+^ | 1222.59 |
| (FPGV)(FPGV) (FPG) | 1.222 | C_58_H_78_N_12_O_11_ | 1119.60 [M+H]^+^ | 1119.88 |
| (FPGV)(FPG)  (FPGV) | 1.592 | C_58_H_78_N_12_O_11_ | 1119.60 [M+H]^+^ | 1119.27 |
| (FPG)(FPGV)  (FPGV) | 1.546 | C_58_H_78_N_12_O_11_ | 1119.60 [M+H]^+^ | 1120.00 |
| (FPGV)(FPG) | 0.466 | C_37_H_50_N_8_O_7_ | 719.39 [M+H]^+^ | 719.01 |
| (FPG)(FPGV) | 0.760 | C_37_H_50_N_8_O_7_ | 719.39 [M+H]^+^ | 719.25 |
| AADAAC-(FPGV)_4_ | 2.496 | C_103_H_145_N_23_O_24_S | 2122.07 [M+H]^+^  2144.05 [M+Na]^+^ | 2122.92  2144.78 |

Retention times of each peptide were determined by RP-UPLC-MS.

Figure S1. UPLC-MS analysis of the synthesized ELP analogues in this study (1/2).

UPLC data of the synthesized peptide are shown. (A) (FPGVG)_5_, (B) (FPGVG)_4_, (C) (FPGV)_5_, (D) (FPGV)_4_, (E) (FPGV)_3_, and (F) (FPGV)_2_. Inset in each panel shows MS spectra of the corresponding peak determined by ACQUITY QDa mass spectrometer. (continue to the next page)

**Figure S1. UPLC-MS analysis of the synthesized ELP analogues in this study (2/2).**

(G) (VFPG)_4_, (H) (FPG)_4_, (I) (FPGV)(FPGV)(FPG), (J) (FPGV)(FPG)(FPGV), (K) (FPG)(FPGV)(FPGV), (L) (FPGV)(FPG), and (M) (FPG)(FPGV). Inset in each panel shows MS spectra of the corresponding peak determined by ACQUITY QDa mass spectrometer.

**
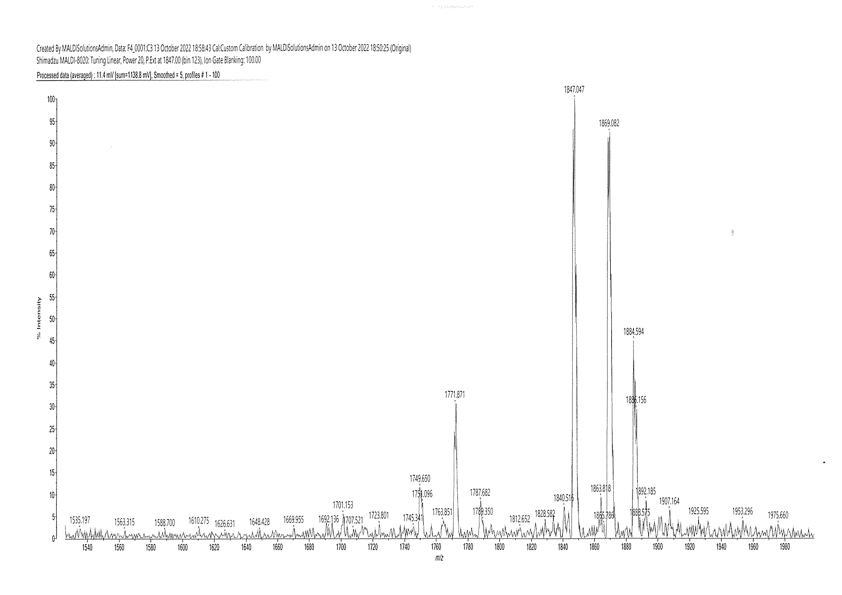

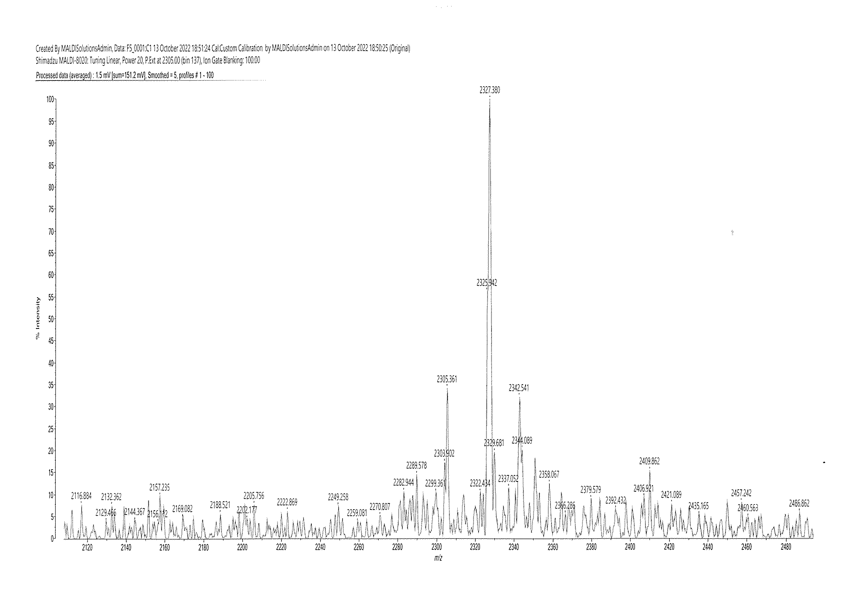
Figure S2. MALDI-TOF MS spectra of the synthesized peptide analogues (1/7).**

(B)

(A)

(A) (FPGVG)_5_, (B) (FPGVG)_4_. (continue to the next page)


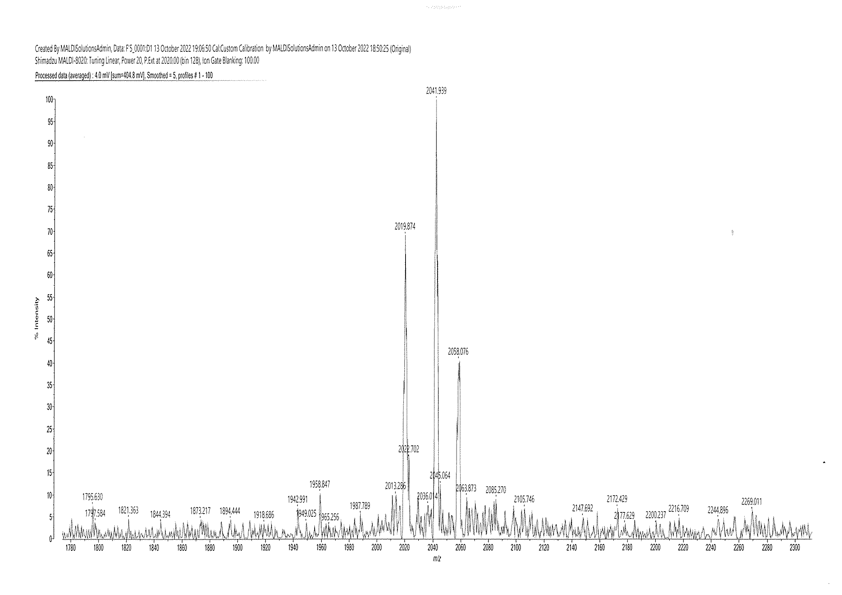

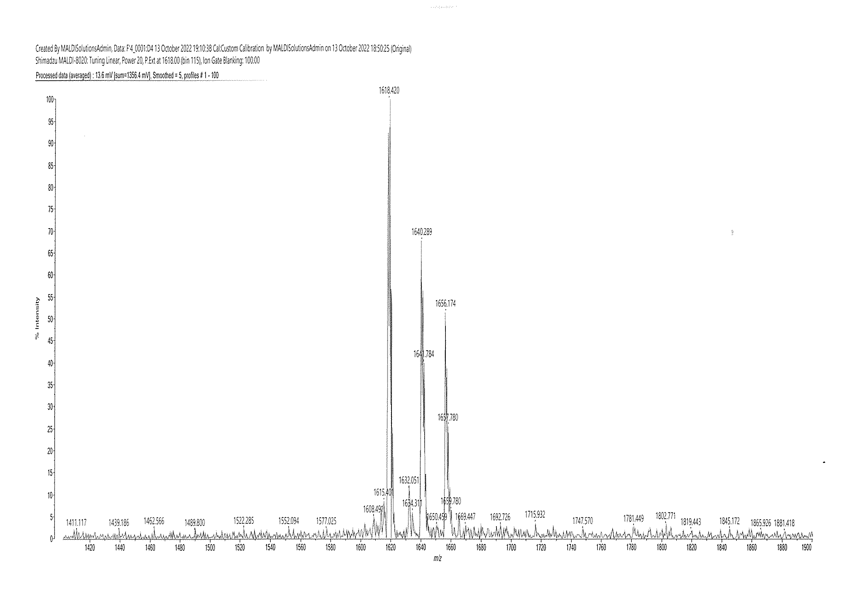
**Figure S2. MALDI-TOF MS spectra of the synthesized peptide analogues (2/7).**

(D)

(C)

(C) (FPGV)_5_, (D) (FPGV)_4_. (continue to the next page)


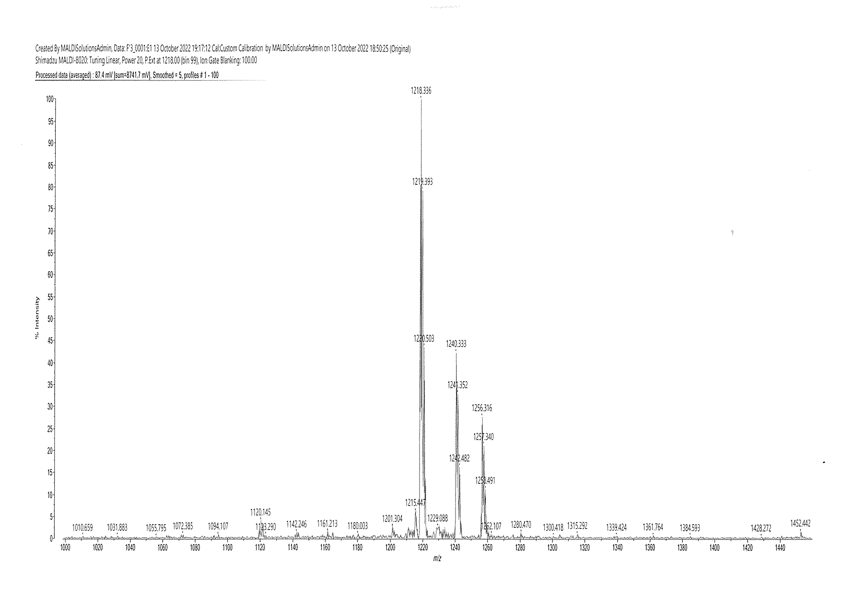

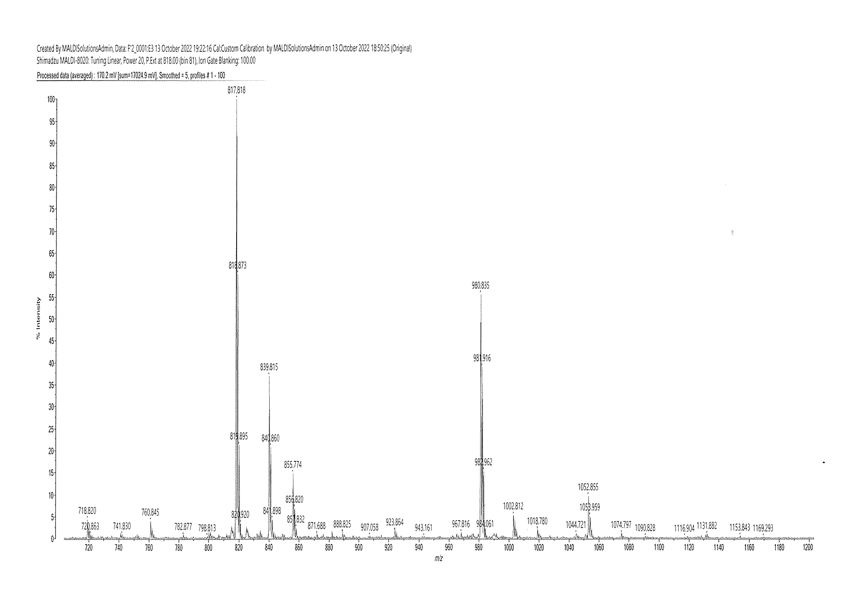
**Figure S2. MALDI-TOF MS spectra of the synthesized peptide analogues (3/7).**

(E)

(F)

(E) (FPGV)_3_, (F) (FPGV)_2_. (continue to the next page)


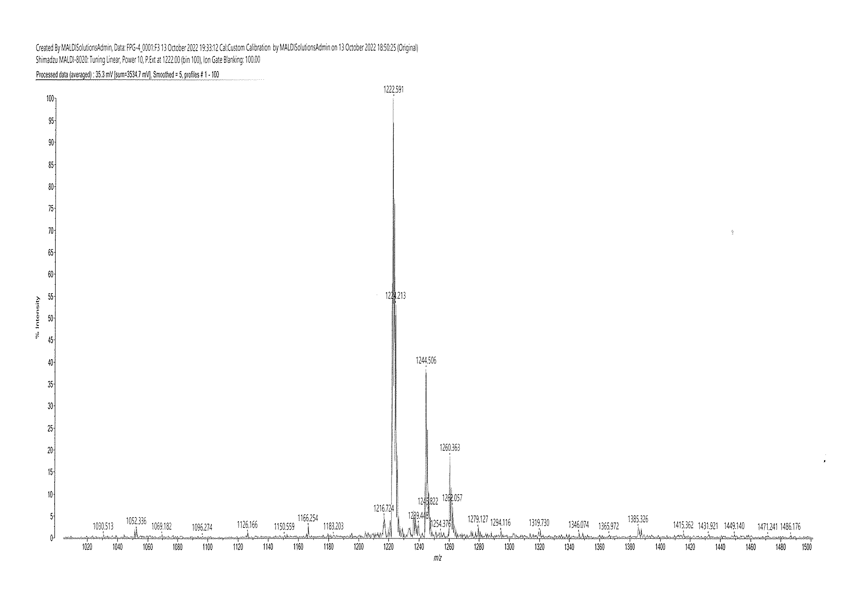

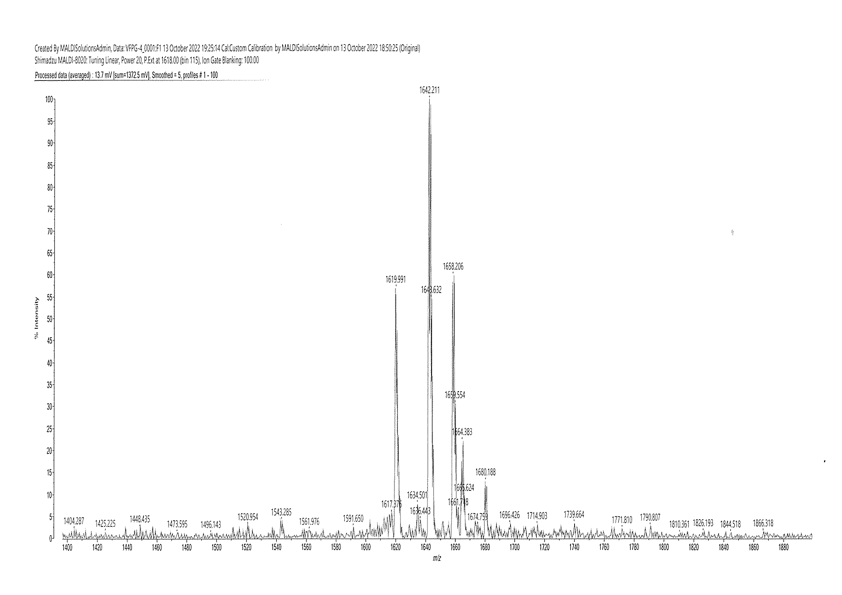
**Figure S2. MALDI-TOF MS spectra of the synthesized peptide analogues (4/7).**

(G)

(H)

(G) (VFPG)_4_, (H) (FPG)_4_. (continue to the next page)

**
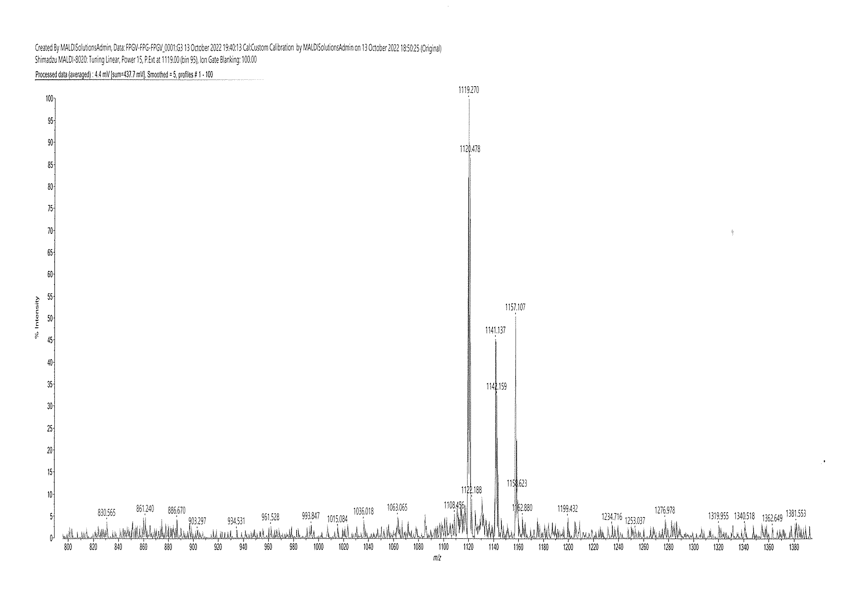

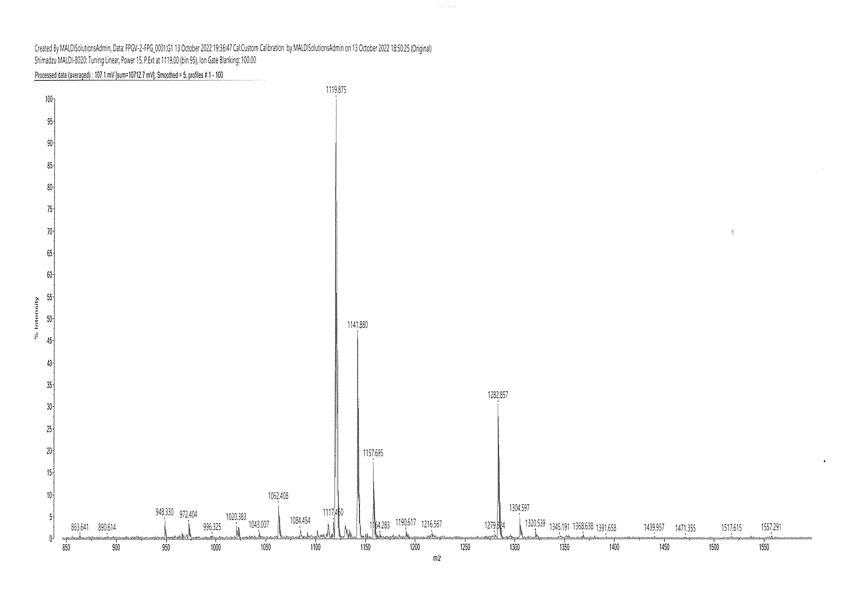
Figure S2. MALDI-TOF MS spectra of the synthesized peptide analogues (5/7).**

(J)

(I)

(I) (FPGV)(FPGV)(FPG), (J) (FPGV)(FPG)(FPGV). (continue to the next page)


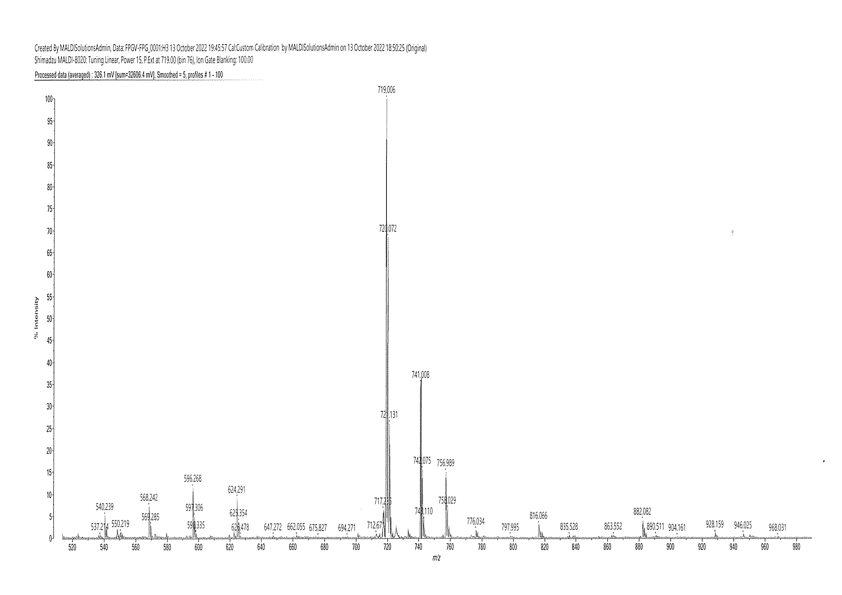

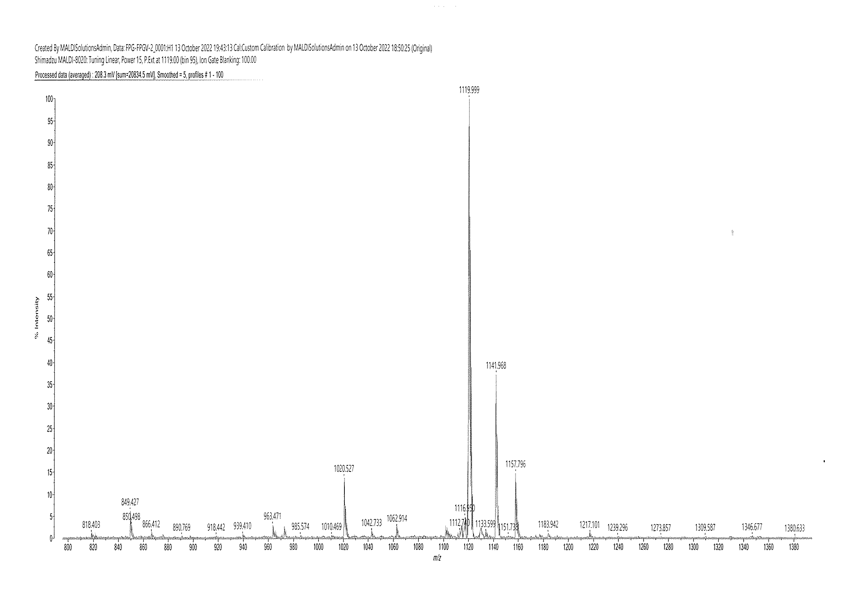
**Figure S2. MALDI-TOF MS spectra of the synthesized peptide analogues (6/7).**

(L)

(K)

(K) (FPGV)(FPGV)(FPGV), (L) (FPGV)(FPG). (continue to the next page)

**
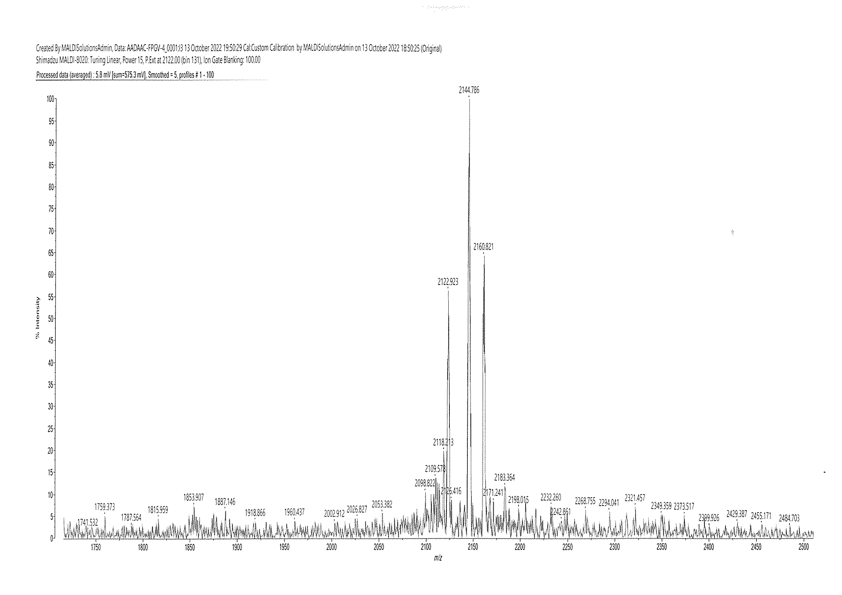

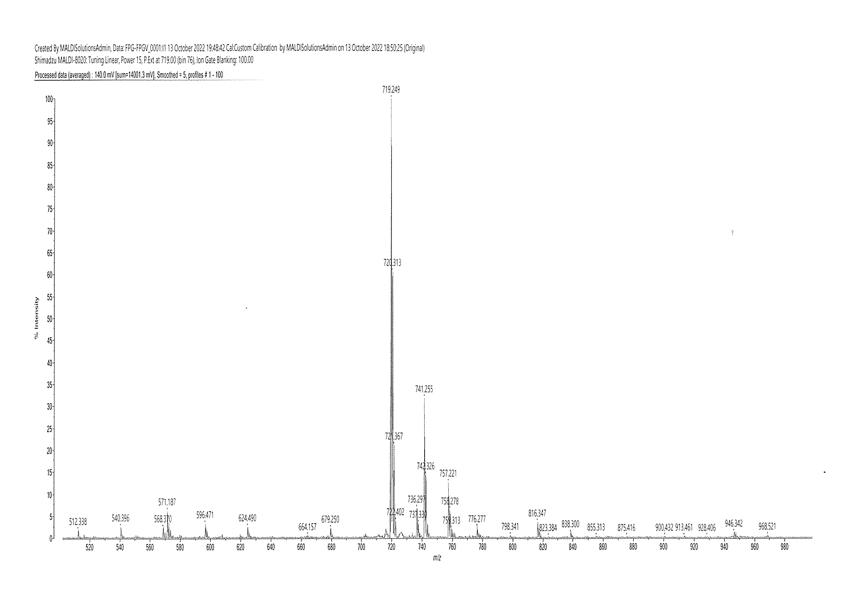
Figure S2. MALDI-TOF MS spectra of the synthesized peptide analogues (7/7).**

(N)

(M)

(M) (FPGV)(FPGV), (N) AADAAC-(FPGV)_4_.

Figure S3. Relationship between the *T*_t_ and molar concentration of truncated ELP analogues.

(FPGVG)_5_ (red line), (FPGV)_5_ (green line), and (FPGV)_4_, (blue line). Each peptide was dissolved in phosphate buffer (27.4 mM Na_2_HPO_4_, 17.8 mM NaH_2_PO_4_, pH 7.4).

**Figure S4. Dynamic light scattering (DLS) autocorrelation curves (1/2).**

(A) (FPGVG)_5_ (10 mg/mL), (B) (FPGVG)_4_ (10 mg/mL), (C) (FPGV)_5_ (2.0 mg/mL), (D) (FPGV)_4_ (10 mg/mL), (E) (FPGV)_3_ (10 mg/mL), and (F) (FPGV)_2_ (30 mg/mL). (continue to the next page)

**Figure S4. Dynamic light scattering (DLS) autocorrelation curves (2/2).**

(G) (VFPG)_4_ (10 mg/mL), (H) (FPGV)(FPGV)(FPG) (20 mg/mL), (I) (FPGV)(FPG)(FPGV) (20 mg/mL), (J) (FPG)(FPGV)(FPGV) (20 mg/mL), (K) (FPGV)(FPG) (50 mg/mL), and (L) (FPG)(FPGV) (50 mg/mL).

Figure S5. Optical microscopy images of (FPGVG)_5_.

The optical microscopic images of (FPGVG)_5_ dissolved in phosphate buffer (27.4 mM Na_2_HPO_4_, 17.8 mM NaH_2_PO_4_, pH 7.4) at a concentration of 10 mg/mL at (A) 5°C and (B) 25°C. The magnification of the images are 40 times. Scale bars indicated 50 μm.

Figure S6. Size distribution analysis of coacervates using optical microscopy images of (FPGVG)_4_.

The binary image of the (FPGVG)_4_ coacervates obtained from Figure 5(B) in the main text. (B) Size distribution of the (FPGVG)_4_ coacervates. Observed particles were classified in 1 µm increments. The number of particles belonging to each class (gray bar) and the average of their diameters (black dashed line) are shown.

Figure S7. Size distribution analysis of coacervates using optical microscopy images of (FPGV)_4_.

The optical microscopic images of (FPGV)_4_ coacervates obtained from Figure 5(D) in the main text. (B) Size distribution of the (FPGV)_4_ coacervates. Observed particles were classified in 1 µm increments. The number of particles belonging to each class (gray bar) and the average of their diameters (black dashed line) are shown.

**
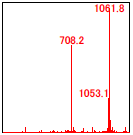
**

Figure S8. Chemical structure and UPLC-MS analysis of AADAAC-(FPGV)_4_.

(A) Chemical structure of AADAAC-(FPGV)_4_ and (B) UPLC-MS analysis of AADAAC-(FPGV)_4_. Inset in (B) shows MS spectra of the corresponding peak determined by ACQUITY QDa mass spectrometer.
